# Exact Two-Component TDDFT with Simple Two-Electron Picture-Change Corrections: X-ray Absorption Spectra Near L- and M-Edges of Four-Component Quality at Two-Component Cost

**DOI:** 10.1021/acs.jpca.2c08307

**Published:** 2023-02-01

**Authors:** Lukas Konecny, Stanislav Komorovsky, Jan Vicha, Kenneth Ruud, Michal Repisky

**Affiliations:** † Hylleraas Centre for Quantum Molecular Sciences, Department of Chemistry, UiT The Arctic University of Norway, N-9037Tromsø, Norway; ‡ Center for Free Electron Laser Science, Max Planck Institute for the Structure and Dynamics of Matter, Luruper Chaussee 149, 22761Hamburg, Germany; § Institute of Inorganic Chemistry, Slovak Academy of Sciences, Dúbravská cesta 9, SK-84536Bratislava, Slovakia; ∥ Centre of Polymer Systems, University Institute, Tomas Bata University in Zlín, CZ-76001Zlín, Czech Republic; ⊥ Norwegian Defence Research Establishment, P.O. Box 25, 2027Kjeller, Norway; # Department of Physical and Theoretical Chemistry, Faculty of Natural Sciences, Comenius University, Ilkovicova 6, SK-84215Bratislava, Slovakia

## Abstract

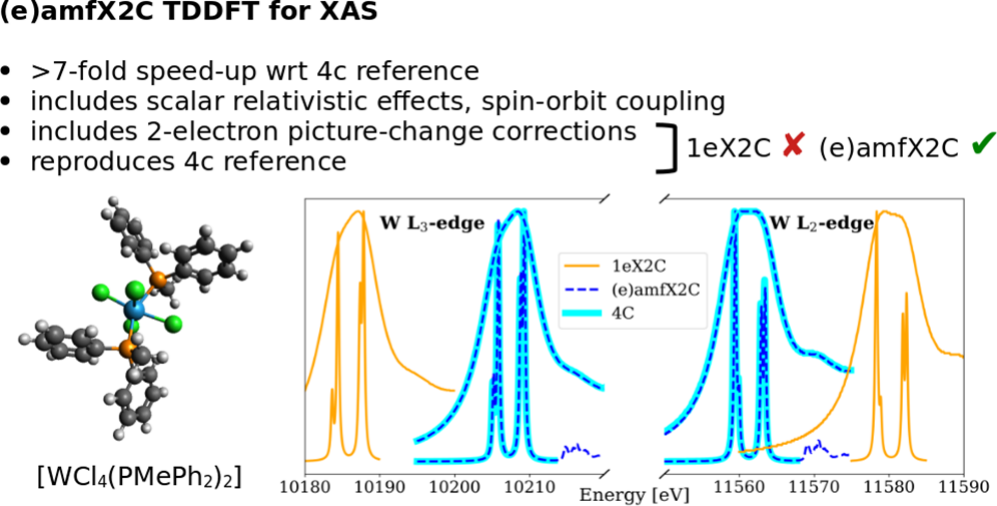

X-ray absorption spectroscopy (XAS) has gained popularity in recent years as it probes matter with high spatial and elemental sensitivities. However, the theoretical modeling of XAS is a challenging task since XAS spectra feature a fine structure due to scalar (SC) and spin–orbit (SO) relativistic effects, in particular near L and M absorption edges. While full four-component (4c) calculations of XAS are nowadays feasible, there is still interest in developing approximate relativistic methods that enable XAS calculations at the two-component (2c) level while maintaining the accuracy of the parent 4c approach. In this article we present theoretical and numerical insights into two simple yet accurate 2c approaches based on an (extended) atomic mean-field exact two-component Hamiltonian framework, (e)amfX2C, for the calculation of XAS using linear eigenvalue and damped response time-dependent density functional theory (TDDFT). In contrast to the commonly used one-electron X2C (1eX2C) Hamiltonian, both amfX2C and eamfX2C account for the SC and SO two-electron and exchange–correlation picture-change (PC) effects that arise from the X2C transformation. As we demonstrate on L- and M-edge XAS spectra of transition metal and actinide compounds, the absence of PC corrections in the 1eX2C approximation results in a substantial overestimation of SO splittings, whereas (e)amfX2C Hamiltonians reproduce all essential spectral features such as shape, position, and SO splitting of the 4c references in excellent agreement, while offering significant computational savings. Therefore, the (e)amfX2C PC correction models presented here constitute reliable relativistic 2c quantum-chemical approaches for modeling XAS.

## 1 Introduction

X-ray absorption spectroscopy (XAS) provides local information about molecular geometry and electronic structure with high elemental specificity due to the energy separation of core levels in different elements.^1^ XAS spectra are conventionally divided into X-ray absorption fine structure (NEXAFS), also known as X-ray absorption near-edge structure (XANES), resulting from excitations of core electrons into vacant bound states, and extended X-ray absorption fine structure (EXAFS) resulting from the excitations into the continuum. While the dependence on large-scale experimental facilities has made X-ray spectroscopy less popular compared to other techniques, advances in instrumentation are changing this trend. More synchrotrons are being built, while at the same time plasma-based and high-harmonic-generation-based tabletop sources are making X-ray spectroscopy more accessible to researchers.^2,3^ We therefore expect an increased interest in XAS and a resulting demand for fast and reliable theoretical methods to predict and interpret the spectra.

The core electronic states probed by X-rays exhibit relativistic effects, both scalar relativistic effects presented as constant shifts and spin–orbit effects that split the core p and d levels into p_1/2_–p_3/2_ and d_3/2_–d_5/2_ levels, respectively. These effects are particularly strong in heavy elements such as transition metals but are measurable already in XAS spectra of third row elements.^4,5^ Therefore, multicomponent relativistic quantum chemical methods with variational inclusion of spin–orbit (SO) coupling present the most reliable approach to these spectral regions.^6^ The “gold standard” in relativistic quantum chemistry is the four-component (4c) methodology including both scalar and SO effects nonperturbatively via the one-electron Dirac Hamiltonian in combination with instantaneous Coulomb interactions among the particles. Since fully 4c calculations of large molecular systems containing heavy elements can be time-consuming, researchers have also focused on the development of approximate two-component (2c) Hamiltonians.^7,8^ The computational advantage of 2c methods over 4c methods comes from discarding the negative-energy states, reducing the original 4c problem by half, and also from abandoning the need to evaluate expensive two-electron integrals over the small-component basis associated with these states. Examples of popular 2c Hamiltonians are the second-order Douglas–Kroll–Hess (DKH2) Hamiltonian,^9−11^ the zeroth-order regular approximation (ZORA) Hamiltonian,^12,13^ and the normalized elimination of small component (NESC) Hamiltonian.^14,15^ A 2c Hamiltonian that has gained wide popularity in recent years is the exact two-component (X2C) Hamiltonian.^16−21^ It reduces the 4c problem to 2c by applying simple algebraic manipulations, avoiding the need to generate explicit operator expressions for (higher-order) relativistic corrections and/or property operators.

There are several flavors of X2C Hamiltonians differing in the *parent* 4c Hamiltonian used to construct a 2c model. The use of a pure one-electron (1e) Dirac Hamiltonian as the parent Hamiltonian results in the one-electron X2C (1eX2C) where two-electron (2e) interactions are entirely omitted from the X2C decoupling transformation step.^8,22^ On the other side is the molecular mean-field X2C approach (mmfX2C)^23^ where the X2C decoupling is performed *after* converged 4c molecular self-consistent field (SCF) calculations, making this approach meaningful only in connection with post-SCF electron correlation and/or property calculations.^23,24^ In between 1eX2C and mmfX2C there exist several parent Hamiltonian models that extend the 1eX2C model by including 2e interactions approximately via (i) element and angular-momentum specific screening factors in the evaluation of 1eSO integrals,^25,26^ (ii) a mean-field SO approach^27^ which has been the basis for the widely popular AMFI module,^28^ and (iii) an approach that exploits atomic model densities obtained within the framework of Kohn–Sham density functional theory (DFT).^29,30^ The screening factors of type i are sometimes referred to as “Boettger factors” or as the screened-nuclear-spin–orbit (SNSO) approach,^31^ obtained from the second-order Douglas–Kroll–Hess DFT-based ansatz.^31^ Its later reparametrization based on atomic four-component Dirac–Hartree–Fock results led to the *modified* SNSO (mSNSO) approach.^32^ Recently, atomic mean-field (amfX2C) and extended atomic mean-field (eamfX2C) approaches have been presented within the X2C Hamiltonian framework,^33^ extending some of earlier ideas of Liu and Cheng^34^ by comprising the full SO and SC corrections that arise from 2e interactions, regardless of whether they arise from the Coulomb, Coulomb–Gaunt, or Coulomb–Breit Hamiltonian. Moreover, this ansatz takes into account the characteristics of the underlying correlation framework, viz., wave function theory or (KS-)DFT, which enables tailor-made exchange–correlation (xc) corrections to be introduced.^33^

The 2c approaches are particularly attractive for X-ray spectroscopies where the typical systems of interest include large transition metal complexes or extended systems such as surfaces and bulk crystals. This is also reflected in DFT being the most popular electronic structure model in XAS calculations since it offers a good balance between efficiency and accuracy.^35^ DFT calculations of XAS spectra can proceed as ΔSCF that subtracts energies of a ground state and a core–hole excited state^36^ or as time-dependent DFT (TDDFT) calculations. The latter can further be approached in three different ways: real-time TDDFT (RT-TDDFT) propagating the electronic density in the time domain,^37,38^ the eigenvalue (Casida) equation based linear response TDDFT (EV-TDDFT),^39,40^ and damped response TDDFT (DR-TDDFT) (also called the complex polarization propagator approach) evaluating the spectral function directly in the frequency domain for the frequencies of interest.^41−43^ RT-TDDFT applied to XAS typically requires simulations with a large number of short time steps to capture the rapid oscillations associated with core-excited states with sufficient accuracy. EV-TDDFT yields infinitely resolved stick spectra in the form of excitation energies and corresponding transition dipole moments but for XAS has to rely on core–valence separation^44−51^ to reach the X-ray spectral region that would otherwise lie too high in the excitation manifold. On the other hand, DR-TDDFT is an efficient way of targeting the XAS spectral function directly for user-defined frequencies even in high-frequency and high density-of-states spectral regions while including relaxation effects and accounting for the finite lifetimes of the excited states by means of a damping parameter.

TDDFT in the relativistic regime is therefore a perspective approach for modeling XAS spectra and has already received some attention in the literature. Previous XAS applications of 4c-DR-TDDFT have been reported in the study of the L_3_-edge of UO_2_^2+^,^52^ as well as XAS of carbon, silicon, germanium, and sulfur compounds.^53^ At the mSNSO X2C level of theory, these include applications of relativistic EV-TDDFT with variational SO interactions,^54^ nonorthogonal configuration interaction,^55^ and the Bethe–Salpeter equation.^56^

The goal of this article is to provide a careful theoretical formulation of the X2C DR- and EV-TDDFT starting from the full 4c time-dependent Kohn–Sham (TDKS) equations. We begin in section 2 by defining the X2C transformation at the level of the TDKS equation. We formulate the X2C-transformed time-dependent Fock matrix at the level of different X2C frameworks, namely the amfX2C, eamfX2C, mmfX2C, and 1eX2C. We introduce the adiabatic X2C approximation where the decoupling matrix is considered static and independent of the external field, allowing us to derive linear response TDDFT in the form of damped response and eigenvalue equations. We conclude the Theory section by summarizing the solvers for DR- and EV-TDDFT and the evaluation of XAS spectra. Section 3 presents the computational details, and section 4 presents the results. First, in section 4.1 a calibration is performed on a series of smaller heavy-metal-containing complexes, comparing the X2C methodologies with reference 4c and experimental data. Then, in section 4.2, amfX2C DR- and EV-TDDFT are showcased on large complexes of chemical interest. Finally, section 5 provides some concluding remarks.

## 2 Theory

Unless stated otherwise, we employ the Einstein summation convention, SI-based atomic units, and an orthonormal atomic orbital (AO) basis indexed by two- or four-component flattened subscripts μ, ν, κ, and λ. Each flattened index accounts for scalar, τ, and multicomponent character, *m* = 2, 4, of relativistic wave functions, e.g., μ ≔ *mτ* (see also ref (57)). Similarly, indices *i*, *j* denote occupied, *a*, *b* denote virtual, and *p*, *q* denote general multicomponent molecular orbitals (MOs), and subscripts *u*, *v* denote Cartesian components. In addition, we indicate matrix and vector quantities by bold and bold-italic fonts, respectively.

A convenient starting point for our discussion of DR-TDDFT in an X2C Hamiltonian framework is to consider the parent four-component (4c) equations of motion (EOM) for occupied molecular orbital coefficients, . Without any loss of generality, we shall consider these equations in the orthonormal basis
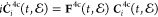
1also because our computer implementation generates the corresponding 2c quantities in such a basis. Equation 1 describes the molecular system in the presence of the time-dependent external electric field , where the time-dependent Fock matrix written in either Hartree–Fock or Kohn–Sham theory has the form
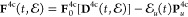
2with  being the time-dependent 4c reduced one-particle density matrix in AO basis
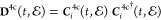
3The coupling of a molecular system to a time-dependent external electric field —the last term on the right-hand side of eq 2—is realized within the dipole approximation by the electric dipole operator −***r***, which is referenced with respect to a gauge origin ***R*** and represented in a 4c basis
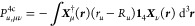
4

Here, **1**_4_ stands for a 4 × 4 unit matrix, and the *orthonormal* four-component μth AO basis function, ***X***_μ_(***r***) ≔ ***X***_μ_^RKB^(***r***), which fulfills the restricted kinetic balance (RKB) condition in its small component part.^58,59^ Note that the interaction of the system with the external electric field described here by the dipole operator is an approximation that can be lifted if necessary.^60,61^ However, we do not focus here on this aspect, assuming the spatial extent of the orbitals involved in the core excitation is much smaller than the wavelength of the incoming radiation, which is valid for metal-to-metal transitions dominating the spectra of the heavy metal complexes considered in the present study. Also note that we work in the length gauge throughout the paper. Finally, we assume a generic form of the electric field, , in the time domain, because its specific formulation is only needed later for the discussion related to response theory.

The field-free Fock matrix **F**_0_^4c^ on the right-hand side of eq 2 characterizes the molecular system of interest in the absence of external fields and consists within the Dirac–Coulomb Hamiltonian framework of the one-electron Dirac contribution **h**^4c^, the two-electron contribution, and the exchange–correlation (xc) contribution
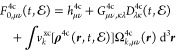
5with *k* = 0, ..., 4. The last term in eq 5 is expressed in terms of the noncollinear xc potential *v*_*k*_^xc^ that is given within a generalized gradient approximation (GGA) by
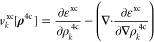
6where ε^xc^ refers to a nonrelativistic xc energy density of GGA type, ρ_*k*_^4c^ represents the 4c electron charge (for *k* = 0) and spin (for *k* = 1, 2, 3) densities
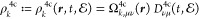
7**Ω**_*k*_^4c^ are overlap distribution functions
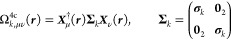
8**σ**_0_≔ **1**_2_, (**σ**_1_, **σ**_2_, **σ**_3_) is a vector constructed from the Pauli matrices, and **1**_2_ and **0**_2_ are 2 × 2 unit and zero matrices, respectively. In this work we use the noncollinear extension of the nonrelativistic xc potentials as described in ref (57) that is based on the noncollinear variables of Scalmani and Frisch.^62^ Note, however, that because we study closed-shell systems in the final response expressions the open-shell *v*_*k*_^xc^ potential reduces to its closed-shell form that only depends on the charge density, and to the closed-shell xc kernel first described in ref (63). The parentheses in eq 6 signify the fact that the gradient operator does not act on the **Ω**_*k*_^4c^ matrix in eq 5. The specific form of the xc contribution to the Fock matrix in eqs 5 and 6 is used here to simplify the following expressions. In practical implementations, however, one uses a formulation that contains **∇Ω**_*k*_^4c^ matrices. This formulation can be obtained from the second term on the right-hand side (RHS) of eq 6 by applying partial integration and employing the fact that both *v*_*k*_^xc^ and **Ω**_*k*_^4c^ vanish as |***r***| → ∞. The two-electron contribution in eq 5 can be written in terms of the matrix of generalized antisymmetrized electron repulsion integrals (ERIs)
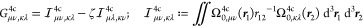
9involving the direct and exact-exchange terms, the latter scaled by a scalar weight factor ζ. Note that the use of **Ω**_0_^4c^(***r***) in ERIs enables us to write an efficient relativistic integral algorithm based on complex quaternion algebra.^59^ Also note that, similarly to ERIs, there is a dependence of the xc contribution on the exact-exchange weight factor ζ for hybrid functionals. However, we do not write this dependence explicitly to simplify the notation.

Before we proceed, let us mention that a convenient way to derive DR-TDDFT equations is to employ an ansatz for the time-dependent MO coefficients^64^
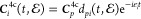
10using the reference (static) 4c MO coefficients ***C***_*p*_^4c^ as the basis, and complex-valued  as expansion coefficients. Both the ***C***_*p*_^4c^ and the corresponding orbital energies ε_*p*_ are obtained from the solution of the time-independent SCF equations^59^
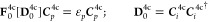
11Finally, applying this ansatz in the EOM for the 4c MO coefficients [eq 1], one obtains within linear response theory the 4c DR-TDDFT expressions as described in ref (64).

### 2.1 Transformation of the EOM to the Exact Two-Component (X2C) Picture

Following the matrix–algebraic approach of X2C, let us assume that, for an arbitrary time and electric field, there exists a unitary transformation matrix  that block-diagonalizes the 4c Fock matrix
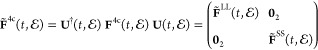
12Note that, to be consistent with our previous work, we use a notation with tildes to indicate picture-change-transformed quantities.^33^ Under this transformation, the parent 4c EOM, eq 1, becomes
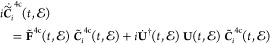
13with
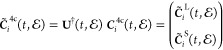
14Without imposing any additional constrains on the unitary transformation, the matrix product  has in the general case nonzero off-diagonal blocks, which prevents a complete decoupling of eq 13. However, this term can be safely neglected within the weak-field and dipole approximations. This statement can be rationalized as follows. The weak-field approximation, , and the electric dipole approximation, ω*lc*^–1^ ≪ 1, where *l* refers to the size of the molecular absorption center, i.e., a small number for the spatially localized excitations considered in this work, leads to the estimate . It follows that , because the matrix  can be estimated as  within the weak-field approximation (see ref (22) and Appendix A for a detailed discussion). As a result, the X2C transformation is approximately constant in time, , which is denoted here as the *adiabatic X2C transformation*, and its use has already been discussed in the context of nonlinear optical property calculations in ref (22). In addition, the field dependence of the X2C unitary transformation can also be safely neglected within the weak-field approximation, because the linear response  is of order  (see Appendix A). As a consequence of the above discussion,  and eq 13 can be written as
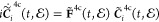
15with an X2C unitary transformation that is both time and electricfield independent, . In the following discussion we employ the simplified notation, **U** ≔ **U**(0, 0).

The best possible transformation matrix **U** which completely decouples the reference positive-energy MOs (+) from those of negative energy (−) can be obtained from the so-called mmfX2C approach.^23^ In the mmfX2C framework, **U** ≔ **U**_mmfX2C_ is obtained *a posteriori* from *converged* time-independent 4c HF/KS equations, eq 11. As a result, the transformed time-dependent MOs [eq 14] become
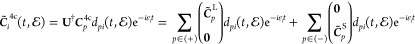
16where **C̃**^L^ ≔ **C̃**_mmfX2C_^L^ and **C̃**^S^ ≔ **C̃**_mmfX2C_^S^. Considering the arguments presented in Appendix B, the negative-energy states [the last term on the RHS of eq 16] contribute to the complex polarizability tensor **α** only of order *c*^–4^. By neglecting this contribution, eq 15 becomes decoupled and one can extract the two-component EOM in the form
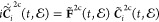
17Here, both the Fock matrix and occupied positive-energy MO coefficients with *i* ∈ (+) are picture-change transformed to 2c form as
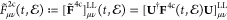
18
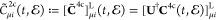
19

### 2.2 Atomic Mean-Field X2C (amfX2C)

As discussed by Knecht et al.^33^ for the static time-independent SCF procedure, the correctly transformed 2c Fock matrix involves the picture-change-transformed density matrix, overlap distribution matrix, and one- and two-electron integrals. One may extend this observation to the time domain as follows:
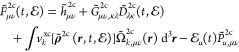
20

The procedure based on eq 20 leads to results equivalent to the 4c ones, but at a computational cost even higher than its 4c counterpart, due to the additional picture-change transformation involved. Therefore, we seek a suitable approximation that enables us to carry out both SCF iterations and linear response calculations in 2c mode such that it is computationally efficient and reproduces the reference 4c results as closely as possible. Keeping this in mind, one can compare eq 20 with an approximate and computationally efficient form of the Fock matrix built with *untransformed* (without the tilde) two-electron integrals **G**^2c^ and overlap distribution matrix **Ω**^2c^, that is
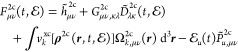
21Here, it is important to emphasize that **ρ**^2c^ also remains untransformed in the sense that an untransformed **Ω**_*k*_^2c^ is used but with the correctly transformed density matrix **D̃**^2c^. We immediately find that the difference between these two Fock matrices expresses the picture-change corrections (PCs) associated with the two-electron integrals and the xc contribution
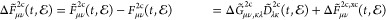
22where *ΔG̃*_*μν*,*κλ*_^2c^ = *G̃*_*μν*,*κλ*_^2c^ – *G*_*μν*,*κλ*_^2c^, and
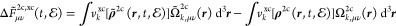
23In line with the work of Knecht et al.,^33^ we exploit the expected local atomic nature of the differential Fock matrix , and approximate it by a superposition of converged atomic quantities rather than the converged molecular one, i.e.
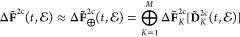
24where *K* runs over all atoms in an *M*-atomic system. Such an approach [eqs 21–24] defines our *atomic mean-field exact two-component* (amfX2C) scheme for the two-electron and xc picture-change corrections applicable to both response and real-time theories. Note that, in contrast to ground-state SCF, where the differential Fock matrix is governed by transformed atomic density matrices that are *static* and *perturbation-independent*,^33^ here the amfX2C scheme also requires that the *time*- and *perturbation*-dependent atomic density matrices are taken into account. These matrices can be expanded to first order in  as
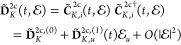
25where the superscripts (0) and (1) indicate the perturbation-free and linear response contributions, respectively [see eq 56]. The zero- and first-order atomic density matrices
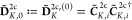
26
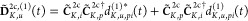
27are obtained from the expansion of  in powers of :
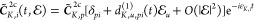
28Here, *d*_*K*,*pi*_^(0)^(*t*) = δ_*pi*_ because we have selected as the starting point for the time evolution of  the reference atomic orbitals that are eigenvectors of the static Fock matrix **F̃**_*K*,0_^2c^[**D̃**_*K*,0_^2c^], .

Our test calculations reveal that, without sacrificing the accuracy of the electric properties, the evaluation of amfX2C PCs by means of eq 24 can be further simplified by considering only zero-order atomic density matrices, that is, by approximating
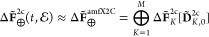
29This scheme leaves the PCs independent of both time and ; i.e., it neglects  terms. In fact, eq 29 defines our approximate amfX2C approach for the two-electron and xc picture-change corrections applicable to both response and real-time theories involving electric fields, and it is used in the response calculations reported in this paper. A pseudocode highlighting the essential steps for evaluating Δ**F̃**_⊕_^amfX2C^ is available in ref (33). With this in mind, the final amfX2C Fock matrix can be written as
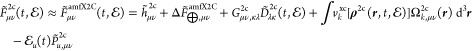
30where  and amfX2C PCs are represented by the time- and perturbation-independent terms Δ**F̃**_⊕_^amfX2C^. Note that the decoupling matrix **U** in the amfX2C approach is obtained by a one-step X2C transformation^20,22^ of the parent 4c Hamiltonian, (**h**^4c^ + **F**_⊕_^4c,2e^), as defined in ref (33).

### 2.3 Extended Atomic Mean-Field X2C (eamfX2C)

The main advantage of the amfX2C approach is that it introduces picture-change corrections to both spin-independent and spin-dependent parts of the two-electron and xc interaction. On the other hand, the fact that these corrections are only introduced in the atomic diagonal blocks of the Δ**F̃**_⊕_^amfX2C^ correction means that, for instance, the direct two-electron contribution will not cancel out with the electron–nucleus contribution at long distances from the atomic centers. This becomes problematic in solid-state calculations, where the exact cancellation of the charge and dipole terms in the expansion at long distances of the direct two-electron and electron–nucleus contributions is essential. In fact, this was the main motivation for introducing an extended amfX2C approach (eamfX2C) at the SCF level.^33^

The generalization of eamfX2C to the time domain requires first to build the 4c molecular density matrix  and its transformed 2c counterpart  from *M* atomic density matrices  and , respectively:
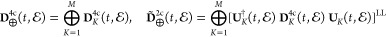
31where all atomic quantities on the RHSs of these equations are represented in *orthonormal* AOs associated with the *K*th atomic center. Then, the molecular density matrices are used to construct the molecular two-electron and exchange–correlation Fock contributions in the full molecular basis, that is
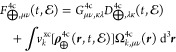
32
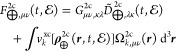
33with
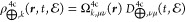
and 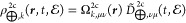
Transforming the 4c Fock matrix [eq 32] and subtracting the approximate 2c Fock matrix [eq 33] lead to our *extended atomic mean-field exact two-component* (eamfX2C) scheme for two-electron and xc picture-change corrections applicable to both response and real-time theories:
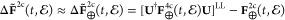
34

Following the aforementioned arguments/estimates for the amfX2C approach, the evaluation of individual atomic density matrices in eq 31 may be approximated in the case of electric properties by their time- and perturbation-independent components. This reduces the eamfX2C PCs in eq 34 to a significantly simpler form
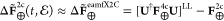
35the evaluation of which can be performed prior to the static SCF as summarized by the pseudocode presented in ref (33). The final picture-change-transformed 2c Fock matrix with PCs represented by eq 35 defines our approximate eamfX2C approach suitable for both response and real-time theories:
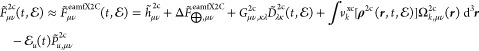
36with . Note that the decoupling matrix **U** in the eamfX2C approach is obtained by a one-step X2C transformation^20,22^ of the parent 4c Hamiltonian, (**h**^4c^ + **F**_⊕_^4c,2e^), as defined in ref (33).

### 2.4 One-Electron X2C (1eX2C) and Molecular Mean-Field X2C (mmfX2C)

The above-mentioned amfX2C and eamfX2C Hamiltonian models for response and real-time theories lie in between two extreme cases. The first one is represented by a pure one-electron X2C (1eX2C) Hamiltonian where two-electron and xc picture-change corrections are omitted entirely. The resulting 1eX2C Fock matrix then reads
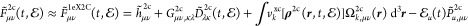
37where the decoupling matrix **U** is obtained simply from the parent one-electron Dirac Hamiltonian.^20,22^ Due to its simplicity the 1eX2C Hamiltonian still remains very popular, but caution is needed when applying this model beyond valence electric properties as shown in this article. The second model, coined as molecular mean-field X2C (mmfX2C),^23^ requires performance of a full molecular 4c SCF calculation in order to determine **U** from converged 4c solutions. The subsequent X2C transformation of the converged 4c Fock matrix gives the mmfX2C Fock matrix:
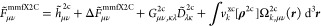
38eigenvalues of which agree up to the computer precision with eigenvalues of positive-energy MOs of the parent 4c Fock matrix. Note that, in eq 38, the two-electron and xc picture-change corrections are included exactly through the differential Fock matrix Δ**F̃**^mmfX2C^ evaluated according to eq 22 (or more precisely by its time- and perturbation-independent counterpart). By following the arguments that justify the use of time- and perturbation-independent (e)amfX2C picture-change -orrection models in response and real-time theories involving electric field(s) [eqs 30 and 36], one can also define a similar model for mmfX2C with the Fock matrix:
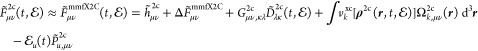
39Here, .

### 2.5 Linear DR-TDDFT within the Presented X2C Approaches

Before we proceed, let us specify the form of the time-dependent electric field  in eqs 30 and 36. In response theory, it is customary to choose the electric field to have the form of a harmonic field of frequency ω and amplitude  that is slowly switched on using the factor η, that is
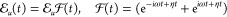
40

Note that the purpose of the field-switching factor η is to ensure a smooth application of the electric field and the limit η → 0 is considered later in the derivation. This is in contrast to the damping factor γ, which describes the rate of the relaxation of the system and should enter the RHS of the EOM in a separate term. However, such a parameter can only enter a relaxation-including equation such as the Liouville–von Neumann equation, an EOM for the density matrix. To simplify the discussion above, we have omitted the factor γ altogether and worked with an EOM for the MO coefficients. Later we add this factor *ad hoc* in eq 42, and we do not discuss the factor η beyond this paragraph. We refer the interested reader to a more detailed discussion of the factors η and γ in refs (64 and 65).

While a direct time propagation of the 2c EOM, eq 17, results in the 2c-RT-TDHF or 2c-RT-TDDFT approaches,^22^ response theory rather seeks for its solution via a perturbation expansion. To this end, we write the expansion of the 2c MO coefficients  in powers of the external field
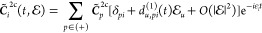
41where *d*_*u*,*pi*_^(1)^(*t*) are the first-order expansion coefficients whose Fourier components are in the end determined within the linear response regime. In eq 41, we have neglected negative-energy states, as discussed below eq 16 and in Appendix B. In addition, we assume  which leads to *d*_*pi*_^(0)^(*t*) = δ_*pi*_. The reference 2c MOs ***C̃***_*i*_^2c^ and one-electron energies ε_*i*_ are eigenvectors and eigenvalues of the static Fock matrix **F̃**_0_^2c^[**D̃**_0_^2c^] ≔ **F̃**^2c^(−∞, 0) [see eqs 30, 36, 37, and 39], respectively. Applying the ansatz in eq 41 to the EOM in eq 17, one can extract the differential equation for the first-order perturbation coefficients **d**_*u*_^(1)^ as follows:
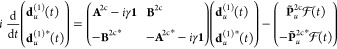
42where **d**^(1)^ and **P̃**_*u*_^2c^are complex matrices of size *N*_v_ × *N*_o_ with *N*_v_ and *N*_o_ referring to the number of 2c virtual and occupied MOs, respectively. Equation 42 is written in terms of the virtual–occupied coefficients *d*_*u*,*ai*_^(1)^ because the occupied–occupied and virtual–virtual ones do not contribute to the time-dependent density matrix (see ref (64) for further details). In eq 42 the matrices **A**^2c^ and **B**^2c^ are defined as
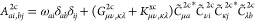
43
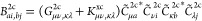
44
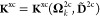
45where no summation is assumed in the first term on the RHS of eq 43, and ω_*ai*_ = ε_*a*_ – ε_*i*_ with ε_*p*_ being the one-electron energy of the *p*th molecular orbital. In eqs 43–45, **G**^2c^ are the 2c untransformed two-electron integrals and **K**^xc^ is the exchange–correlation kernel constructed from 2c untransformed overlap distribution functions **Ω**_*k*_^2c^ and the transformed 2c density matrix **D̃**^2c^. The functional form of the noncollinear xc kernel follows the one presented by Bast et al.;^63^ however, in this work we utilize the RKB basis in contrast to the unrestricted kinetic balance basis employed in ref (63).

The differential equation in eq 42 can be turned into an algebraic form by the method of undetermined coefficients, substituting
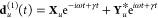
46where **X**_*u*_ and **Y**_*u*_ are complex matrices of time-independent undetermined coefficients. After substituting eq 46 into eq 42 and collecting terms proportional to e^–*iωt*+*γt*^, one arrives at the final linear damped response equation:
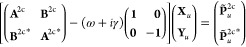
47Both ω and γ are user-defined parameters specifying the external electric field frequency and a common relaxation (damping) parameter modeling the finite lifetime of the excited states that leads to finite-width peaks. The right-hand side of eq 47 describes the interaction of the molecular system with the applied external electric field, which in the electric dipole approximation is mediated by the electric dipole moment operator.

In addition, the solution of the homogeneous form of the linear system of differential equations in eq 42, i.e., for , leads to the eigenvalue TDDFT (EV-TDDFT) equation
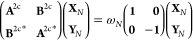
48where ω_*N*_ represents a vertical electronic excitation energy from the reference state to the *N*th excited state with a transition vector (**X**_*N*_**Y**_*N*_)^T^. This equation represents another linear response TDDFT approach to molecular properties such as XAS spectra. Note that despite the similarity in notation, the response vector (**X**_*u*_**Y**_*u*_)^T^ of the damped response TDDFT and the transition vector (**X**_*N*_**Y**_*N*_)^T^ of the eigenvalue TDDFT have different meanings and units. While the former describes the response to an external perturbation and depends on its operator, frequency, and damping factor, the latter only describes the transition amplitude between the ground state and *N*th excited state.

DR-TDDFT, eq 47, is solved using an iterative subspace algorithm, since the size of the matrix on the left-hand side of the equation prohibits its direct inversion or the use of elimination techniques for realistic molecular systems. Because the equation has the same properties in terms of complexity and symmetries as the 4c DR-TDDFT equation, we can employ the same solver as is used for the 4c case.^64,66^ The iterative subspace solver implemented in the ReSpect program^59^ explicitly treats the terms in the response equation based on their hermicity and time-reversal symmetry and allows several frequencies (tens to hundreds) to be considered simultaneously, thus covering a large part of the spectrum in a single run. A detailed presentation of this solver is available in ref (64). Similarly, the eigenvalue linear response TDDFT equation, eq 48, is also solved iteratively by a variant of the Davidson–Olsen algorithm, as presented in ref (57).

The calculation of XAS spectra in the linear response regime corresponds to evaluating the dipole strength function
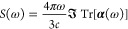
49where *c* is the speed of light,  denotes the imaginary part, Tr is the trace over the Cartesian components, and **α**(ω) is the complex polarizability tensor in the frequency domain. This tensor parametrizes the first-order electric dipole response, i.e., the induced electric dipole moment **μ**^ind^(ω), to an external electric field as
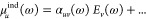
50In 2c-DR-TDDFT, the **α**(ω) tensor components are calculated for a user-defined set of frequencies from the response vector (**X**_*u*_**Y**_*u*_)^T^, the solution of eq 47, via
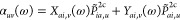
51In contrast, the evaluation of the complex polarizability tensor from the solution (**X**_*N*_**Y**_*N*_)^T^ of the EV-TDDFT equation, eq 48, proceeds via a calculation of the transition dipole moment
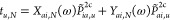
52which is then inserted into the expression for the polarizability as a linear response function
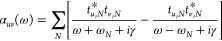
53

Here, the frequency ω and damping parameter γ are included in the calculation of α_*uv*_(ω), i.e., essentially in a postprocessing step, while in DR-TDDFT they are terms in the main working equation, eq 47. This difference has consequences for the workflow and practicality of the DR-TDDFT and eigenvalue TDDFT in various situations, even though the methods yield identical final spectra assuming the same γ factor is used.

## 3 Computational Details

For the purpose of benchmarking and calibration, a set of closed-shell heavy-metal-containing compounds with high-quality experimental data available, including 3d, 4d, 5d, and 5f elements with various electron configurations of the central atom, was selected, specifically VOCl_3_, CrO_2_Cl_2_, MoS_4_^2–^, WCl_6_, PdCl_6_^2–^, ReO_4_^–^, and UO_2_(NO_3_)_2_. Moreover, XAS spectra of larger systems, namely [RuCl_2_(DMSO)_2_(Im)_2_] (Im = imidazole, DMSO = dimethyl sulfoxide), [WCl_4_(PMePh_2_)_2_] (Ph = phenyl), and [(η^6^-*p*-cym)Os(Azpy-NMe_2_)I]^+^ (*p*-cym = *p*-cymene, Azpy-NMe_2_ = 2-(*p*-([dimethylamino]phenylazo)pyridine)), are included. Geometries were optimized using the TURBOMOLE quantum-chemical program^67^ with a protocol designed for transition metal elements:^68,69^ PBE0 functional,^70−73^ def2-TZVPP basis sets^74^ for all atoms (def-TZVP for uranium complex) with corresponding effective core potentials (ECPs)^75^ replacing 28 core electrons in 4d and 60 electrons in 5d and 5f elements.

All X-ray spectra were calculated using the damped response library^64^ and eigenvalue linear response TDDFT library^57^ of the relativistic spectroscopy DFT program ReSpect.^59^ Uncontracted all-electron GTO basis sets were used for all systems. The selected basis sets were the uncontracted Dyall's VDZ basis sets^76−81^ for metals and iodine (basis sets for 3d elements are available upon request) and the uncontracted Dunning's aug-cc-pVDZ basis sets^82−84^ for light elements. The systems were treated using the PBE0 density functional including a modified version, PBE0-*x*HF, with variable exact-exchange admixture *x* that was previously shown to be crucial to counter the shifts observed with standard parametrizations.^85^ The numerical integration of the noncollinear exchange–correlation potential and kernel was done with an adaptive molecular grid of medium size (program default). In the 2c calculations, atomic nuclei of finite size were approximated by a Gaussian charge distribution model.^86^

The damped linear response calculations covered the spectral regions with a resolution of 0.1 eV. The initial guesses of the spectral regions to be scanned were provided by the orbital energies of the target core orbitals. Core–valence separation^44−51^ was used to remove nonphysical valence-to-continuum excitations that may occur at the same energy ranges as the physical core excitations. All damped response calculations employed the multifrequency solver with 100 frequencies treated simultaneously. The damping/broadening parameter used in the damped response calculations was set to 0.15 eV for high-resolution spectra, while values ranging from 0.5 to 3 eV were used to obtain wider peaks to facilitate the comparison with experimental line shapes. Since the value of the damping parameter γ affects the amplitude of the spectra, in graphs where we compare spectra with different damping parameters or with normalized experimental spectra, we normalize the calculated spectra to unity. This is denoted by arbitrary units (arb. units) instead of atomic units (au) as the dimension of the spectral function.

The eigenvalue linear response TDDFT calculations used core–valence separation to access excitation energies associated with core-excited states. The eigenvalue equation was solved iteratively for the first 50 excitation energies. The spectra were subsequently calculated from the excitation energies and transition moments obtained from the eigenvectors with the same Lorentzian broadening as in the corresponding DR-TDDFT calculations.

## 4 Results and Discussion

### 4.1 Calibration of X2C Approaches

**Figure 1:**
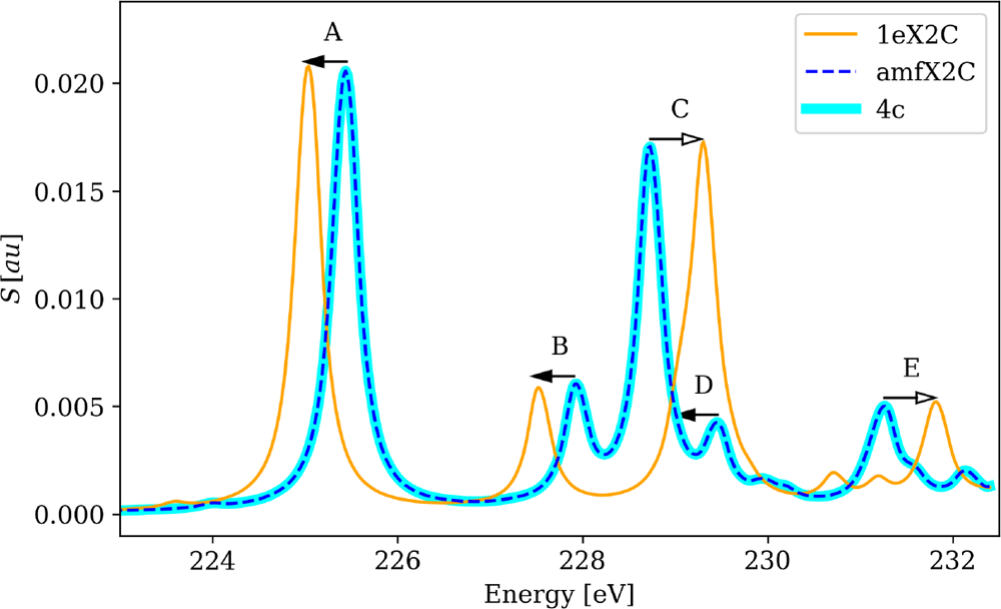
amfX2C Hamiltonian reproduces the 4c reference while 1eX2C Hamiltonian overestimates spin–orbit splitting leading to incorrect 1eX2C spectral shape of XAS spectra of MoS_4_^2–^ near Mo M_4,5_-edges calculated using PBE0 functional and DZ/aDZ basis sets.

**Figure 2:**
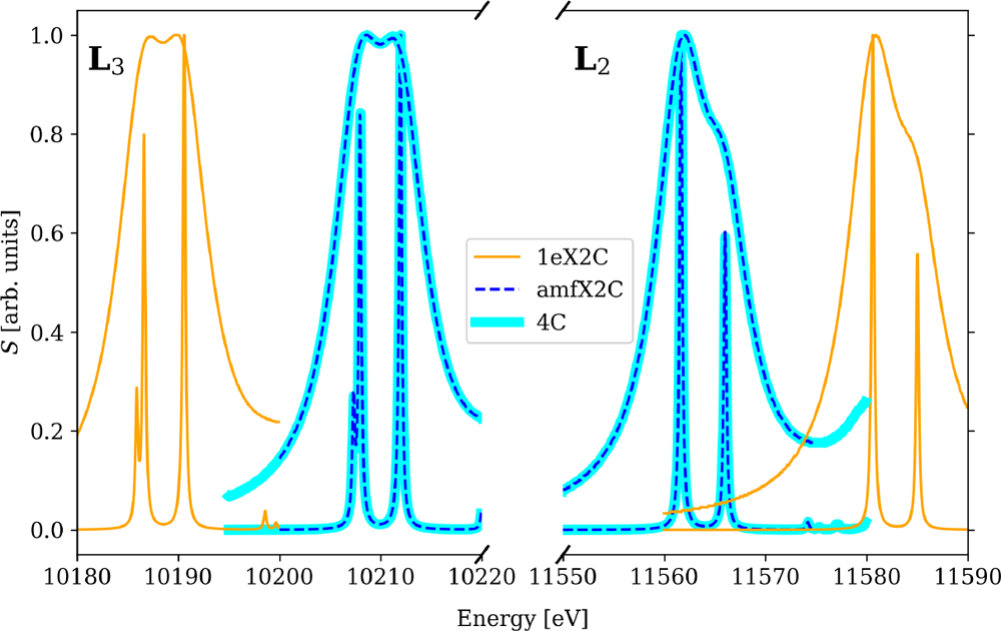
amfX2C Hamiltonian reproduces the 4c reference while 1eX2C Hamiltonian overestimates spin–orbit splitting leading to 20 eV shifts in XAS spectra of WCl_6_ near W L_2,3_-edges calculated using PBE0-60HF functional and DZ/aDZ basis sets. Broad peaks were obtained with damping parameter γ = 3.0 eV, while narrow ones were obtained with γ = 0.15 eV.

To determine the accuracy of the developed X2C DR-TDDFT approaches, we first repeated the calibration study from our previous work focused on the 4c method.^85^ The calibration set consists of XAS spectra near L_2,3_- and M_4,5_-edges of various 3d, 4d, 5d, and 5f elements in small molecules. For these we determined offsets from experimental values using 1eX2C, amfX2C, and mmfX2C relativistic DR-TDDFT and compared these offsets to the 4c results. The results are summarized in Table 1. We see that the one-electron X2C variant (1eX2C) overestimates the spin–orbit splitting with respect to the 4c calculations as well as to the experiment. This error can be attributed to not accounting for the transformation of the two-electron term in the one-electron X2C approximation. This shortcoming is remedied in the amfX2C and mmfX2C approaches, which exactly reproduce the 4c spectral line positions. The agreement holds for the whole spectra as seen in Figures 1 and 2, demonstrating the shortcomings of 1eX2C and the improvements achieved by amfX2C and mmfX2C. The eamfX2C method gives the same results as amfX2C as demonstrated in Table S1. However, in the rest of the paper, we focus on the computationally simpler amfX2C approach.

**Table 1:** Main Line Positions and Spin–Orbit Splittings in XAS Spectra Calculated Using DR-TDDFT with PBE0 and PBE0-*x*HF Functionals in Dyall-DZ/aDZ Basis Sets at the X2C Relativistic Level of Theory (1e, (e)amf, mmf) Compared with Fully Relativistic 4c Results and Experimental Data^a^

			PBE0-25HF				PBE0-*x*HF				
		expt^b^	4c	1eX2C	(e)amfX2C	mmfX2C	*x*HF	4c	1eX2C	(e)amfX2C	mmfX2C
VOCl_3_	L_3_	516.9	507.5	507.1	507.5	507.5	50	515.2	514.9	515.2	515.2
	L_2_	523.8	514.2	514.5	514.2	514.2	50	522.0	522.3	522.0	522.0
	ΔSO	6.9	6.7	7.4	6.7	6.7		6.8	7.4	6.8	6.8
CrO_2_Cl_2_	L_3_	579.9	571.4	571.0	571.4	571.4	50	580.0	579.6	580.0	580.0
	L_2_	588.5	579.5	579.8	579.5	579.5	50	588.2	588.5	588.2	588.2
	ΔSO	8.6	8.1	8.8	8.1	8.1		8.2	8.9	8.2	8.2
MoS_4_^2–^	L_3_	2521.7	2489.3	2486.2	2489.3	2489.3	60	2523.1	2517.8	2523.1	2523.1
	L_2_	2626.0	2595.9	2597.8	2595.9	2595.9	60	2627.6	2630.3	2627.6	2627.6
	ΔSO	104.3	106.6	111.6	106.6	106.6		104.5	112.5	104.5	104.5
	M_5_	228.7	225.4	225.0	225.4	225.4	40	228.9	228.5	228.9	228.9
	M_4_	231.7	228.7	229.3	228.7	228.7	40	232.2	232.8	232.2	232.2
	ΔSO	3.0	3.3	4.3	3.3	3.3		3.3	4.3	3.3	3.3
PdCl_6_^2–^	L_3_	3177.8	3138.2	3133.9	3138.2	3138.2	60	3173.4	3169.3	3173.4	3173.4
	L_2_	3334.7	3297.4	3300.1	3297.4	3297.4	60	3332.9	3336.5	3332.9	3332.9
	ΔSO	156.9	159.2	166.2	159.2	159.2		159.5	167.2	159.5	159.5
WCl_6_	L_3_	10212.2	10139.8	10117.5	10139.9	10139.8	60	10207.3	10185.9	10207.3	10207.3
	L_2_	11547.0	11492.8	11505.9	11492.7	11492.8	60	11561.7	11580.6	11561.6	11561.7
	ΔSO	1334.8	1353.0	1388.4	1352.8	1353.0		1354.4	1394.7	1354.3	1354.4
ReO_4_^–^	L_3_	10542.0	10472.0	10448.2	10471.7	10472.0	60	10541.0	10518.6	10541.0	10541.0
	L_2_	–	11911.9	11925.9	11911.9	11911.9	60	11982.1	12002.1	11982.1	11982.1
	ΔSO	–	1439.9	1477.7	1440.2	1439.9		1441.1	1483.5	1441.1	1441.1
UO_2_(NO_3_)_2_	M_5_	–	3515.3	3504.5	3515.3	3515.3	60	3549.9	3539.2	3549.9	3549.9
	M_4_	3727.0	3693.2	3704.7	3693.2	3693.2	60	3728.0	3740.5	3728.0	3728.0
	ΔSO	–	177.9	200.2	177.9	177.9		178.1	201.3	178.1	178.1

a The amf and eamf Hamiltonians give identical results; see Table S1.

b Experimental references: VOCl_3_, ref (91); CrO_2_Cl_2_, ref (91); MoS_4_^2–^, ref (92); WCl_6_, ref (93); PdCl_6_^2–^, ref (94); ReO_4_^–^, ref (95); UO_2_(NO_3_)_2_, ref (96).

While the overestimation of the SO splitting by 1eX2C is a *quantitative* effect of shifting the excitation energies, in calculations of the whole spectra, it can also be manifested as a *qualitative* change of spectral shapes. This happens particularly in cases when edges separated by SO splitting (L_2_–L_3_, M_2_–M_3_, M_4_–M_5_) overlap, and peaks from both edges fall into the same energy window. This effect is illustrated for the spectra near the molybdenum M_4_- and M_5_-edges of MoS_4_^2–^ (PBE0 functional, DZ/aDZ basis sets) depicted in Figure 1. While amfX2C reproduces the reference 4c results exactly, 1eX2C overestimates the SO splitting causing a red shift of the M_5_ lines and a blue shift of the M_4_ lines. Namely, lines A, B, and D belonging to the M_5_-edge are in the 1eX2C description shifted to lower energies (full arrows), while lines C and E belonging to the M_4_-edge are shifted to higher energies (empty arrows). As a consequence, lines C and D overlap and merge in the 1eX2C spectrum with the given broadening parameter, resulting in a different overall spectral shape when compared to 4c and amfX2C data.

Since amfX2C, eamfX2C, and mmfX2C reproduce the 4c spectra, they allow the same computational protocol developed in the context of 4c calculations to be reused with these 2c Hamiltonians. The protocol aims to reproduce experimental spectral line positions to avoid arbitrary shifting of calculated spectra and is based on increasing the admixture of Hartree–Fock exchange (HFX) in hybrid functionals, with the optimal value being 60% above 1000 eV independent of the underlying pure xc potential, e.g., PBE0 or B3LYP.^73,87−90^ This is contrasted with 1eX2C, whose overestimation of the spin–orbit coupling is also present in the calculations with increased HFX (see Figure 2). As a result, the spectra calculated with the HFX amount determined at the 4c level of theory miss the experimental edge positions. 1eX2C would thus need its own computational protocol that would have to rely on error cancellation. However, using amfX2C, eamfX2C, and mmfX2C, we can reproduce the experimental data at the two-component relativistic level of theory in the same way as at the 4c level; see the last four columns of Table 1 and Table S1.

A motivation for the development of two-component methods is their lower computational cost. To determine the performance of the X2C DR-TDDFT in this regard, a comparison of computational times required for a single iteration of DR-TDDFT calculations is reported in Table 2 along with times required for SCF iterations for the systems considered in the benchmark study as well as one of the larger complexes. Here we consider DR-TDDFT calculations for a single frequency point rather than for 100 frequencies treated together (the setup used in the other calculations in this paper). This choice is made to allow for an equivalent comparison between the 2c and 4c levels of theory, since the details of the multifrequency implementations in our program differ between these cases. Therefore, these times serve only as a measure of the speedup achieved by X2C DR-TDDFT against 4c DR-TDDFT rather than of the total computational cost of the calculations.

**Table 2:** Computational Cost of One Iteration of SCF and DR-TDDFT Calculations^a^

		time		
system	step	4c	amfX2C	speedup
VOCl_3_	SCF	9.2 s	2.6 s	3.5
	DR-TDDFT	43.4 s	6.5 s	6.7
CrO_2_Cl_2_	SCF	7.7 s	2.3 s	3.3
	DR-TDDFT	37.9 s	5.8 s	6.5
MoS_4_^2–^	SCF	13.2 s	4.2 s	3.1
	DR-TDDFT	52.4 s	8.4 s	6.2
PdCl_6_^2–^	SCF	29.6 s	9.3 s	3.2
	DR-TDDFT	1 min 46.7 s	16.7 s	6.4
WCl_6_	SCF	59.2 s	17.2 s	3.4
	DR-TDDFT	3 min 0.3 s	26.4 s	6.8
ReO_4_^–^	SCF	23.5 s	6.6 s	3.6
	DR-TDDFT	1 min 33.8 s	13.1 s	7.2
UO_2_(NO_3_)_2_	SCF	2 min 54.1 s	47.2 s	3.7
	DR-TDDFT	8 min 44.3 s	59.9 s	8.8
[WCl_4_(PMePh_2_)_2_]	SCF	1 h 15 min 2.2 s	24 min 31.0 s	3.1
	DR-TDDFT	4 h 19 min 1.3 s	37 min 23.1 s	6.9

a The calculations were performed on a single computer node equipped with an Intel Xeon-Gold 6138 2.0 GHz processor with 40 CPU cores. The Intel ifort 18.0.3 compiler with -O2 optimization and the parallel Intel MKL library were used for compilation and linking.

An iteration of a subspace solver of relativistic X2C as well as 4c DR-TDDFT is dominated by the calculation of the two-electron integrals that are needed to obtain the elements of matrices **A**^2c^ and **B**^2c^ in eq 47. These matrix elements are on-the-fly contracted with the elements of the so-called trial vectors that constitute the basis of the subspace in which the solution (**X**_*u*_**Y**_*u*_)^T^ is sought (see the algorithm in ref (64)). Since the trial vectors are constructed with defined Hermitian and time-reversal symmetries, only those elements of matrices **A**^2c^ and **B**^2c^ giving nonzero contributions are calculated owing to the efficient quaternion implementation. The calculations were performed on a single computer node equipped with an Intel Xeon-Gold 6138 2.0 GHz processor with 40 CPU cores. The Intel ifort 18.0.3 compiler with -O2 optimization and the parallel Intel MKL library were used for compilation and linking. The times show an approximately 7-fold speedup achieved by amfX2C across the systems, which is similar to the performance reported in our earlier work for real-time 1eX2C TDDFT,^22^ while the speedup in the SCF step is also in line with previous results.^59^ The acceleration achieved by amfX2C together with its accuracy thus pushes the boundaries of relativistic XAS calculations toward larger and experimentally more relevant systems.

There are several factors affecting total calculation times such as the number of iterations needed for convergence and the number of trial vectors generated in one iteration. For a given molecular system and basis set, these depend on (i) the chosen frequency range—spectrally dense regions require more trial vectors and iterations; (ii) the damping parameter γ—calculations with smaller γ’s require more trial vectors and iterations; (iii) the method for isolating core excitations—the technique based on zeroing elements of the perturbation operator used in our previous work^85^ is more demanding than core–valence separation used in this work, since additional (small-amplitude) elements of the response vectors need to be converged as well.

With these caveats in mind, let us examine total calculation times required to obtain XAS spectra of [WCl_4_(PMePh_2_)_2_] utilizing PBE0-60HF functional and DZ/aDZ basis sets. Here, we used the same hybrid-parallel computational setup as in our previous reference 4c calculations,^85^ utilizing 16 computer nodes each equipped with AMD Epyc 7742 2.25 GHz processors with 128 CPU cores. The hybrid parallelization facilitates OpenMPI library (version 4.0.3) with 8 MPI processes per node and 16 OMP threads per MPI process. The compilation was done using Intel ifort 19.1.1.217 compiler with -O2 optimization and linked to the in-build OMP-parallel Intel MKL library. For the case of high density of states regions, namely 11560–11570 eV (W L_2_-edge) and 10205–10215 eV (W L_3_-edge), each comprising 100 frequency points, the 4c calculations with γ = 3.0 eV solving the full DR-TDDFT equation with elements of the perturbation matrix outside the core–virtual orbital pairs set to zero lasted approximately 85 h 49 min and 88 h 59 min, respectively. Equivalent amfX2C calculations requiring similar numbers of iterations (±1) and trial vectors took approximately 7 h 33 min (speedup 11.4) and 8 h 36 min (speedup 10.3). The use of CVS in amfX2C brought the CPU times down to 1 h 39 min and 2 h 8 min mainly by decreasing the total number of iterations and trial vectors. As a rule of thumb, we can therefore conclude that a calculation that would have previously taken a week can now be finished in less than a day while utilizing the same computational resources without the loss of accuracy.

### 4.2 Larger Systems

The main goal in the development of X2C-based EV- and DR-TDDFT is to allow multicomponent relativistic calculations to be applied to large systems of chemical interest, such as heavy-metal-containing complexes with complicated and heavy-atom-containing ligands. In this section we report spectra of such systems. Of the three X2C approaches, amfX2C, eamfX2C, and mmfX2C, that reproduced the 4c data in the calibration presented in section 4.1, we focus in the following on amfX2C. This is because, in amfX2C, the whole calculation including the initial ground-state SCF is performed in a two-component regime and in a simpler way than in eamfX2C. That is why we envision amfX2C to become the standard method of choice in future relativistic calculations of XAS spectra of large molecular systems.

**Figure 3:**
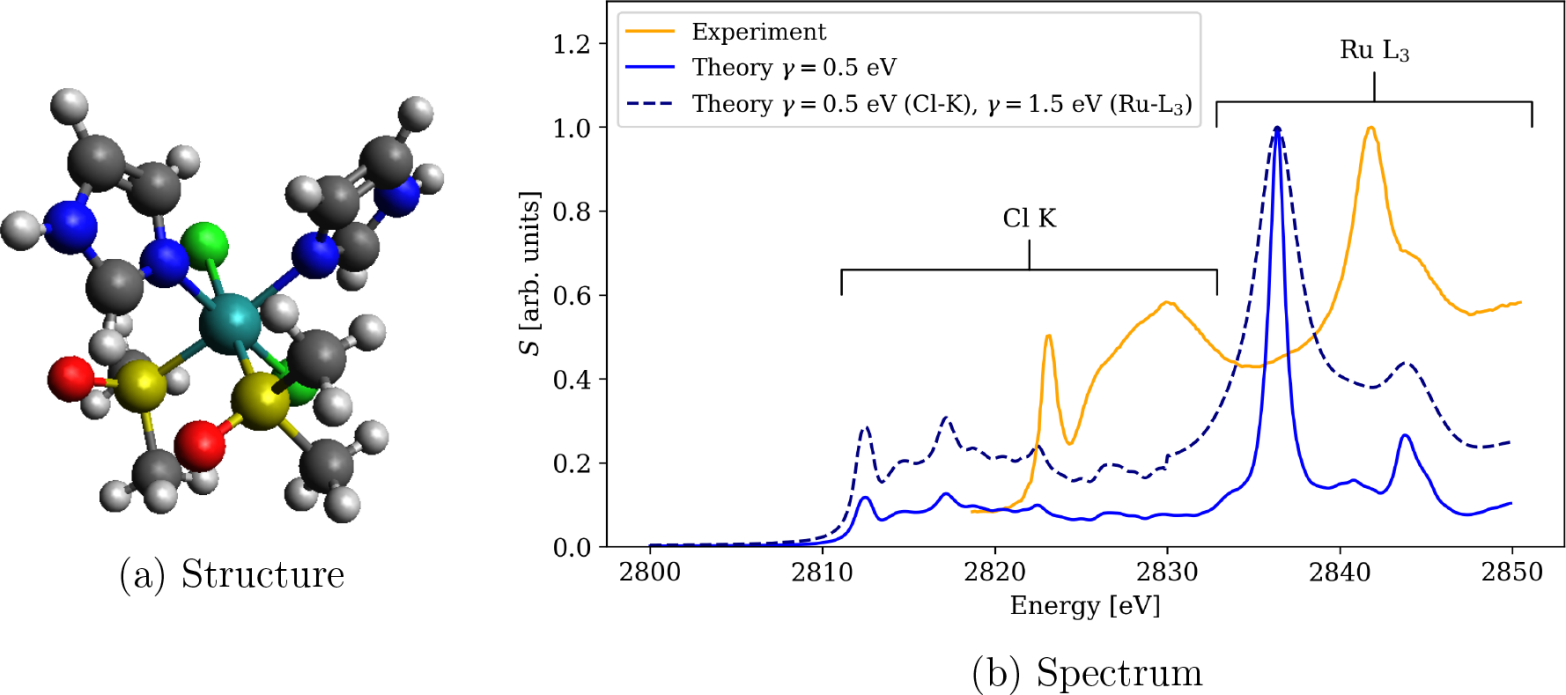
XAS spectrum of [RuCl_2_(DMSO)_2_(Im)_2_] near overlapping Cl K-edge and Ru L_3_-edge calculated using PBE0-60HF functional and DZ/aDZ basis sets. Both absorption edges are reproduced sufficiently well when different inverse lifetimes (γ) of core-excited states of different atoms are accounted for. No shift was applied on the energy axis to manually align the spectra. Experimental spectrum digitized from ref (98).

First, for [RuCl_2_(DMSO)_2_(Im)_2_] (Figure 3a) we calculated the spectra in the region 2800–2850 eV, covering both Cl K-edge and Ru L_3_-edge (Figure 3b). The spectra are in general well aligned with experiment with differences of 11 and 5.5 eV in the Cl K-edge and Ru L_3_-edge positions, respectively, corresponding to a slight, 5.5 eV, overestimation of the separation between the edges. In addition, as noted before,^85^ the position of lines within the same absorption edge increases with the amount of HFX in the functional, resulting in a somewhat wider L_3_-edge peak in the calculation. While we previously reported spectra calculated with a single damping parameter, γ = 0.5 eV, for the whole spectral range, here we also performed calculations with a larger damping parameter, γ = 1.5 eV, in the region near the Ru L_3_-edge, corresponding to a shorter lifetime of the Ru p_3/2_ excited state. Since this damping parameter was determined from the experimental spectrum, it led to a better agreement with the experimental reference. This supports an idea also suggested in our previous work^85^ for a computational protocol utilizing different damping parameters in complex polarization propagator (CPP) calculations of XAS spectra near overlapping edges.

**Figure 4:**
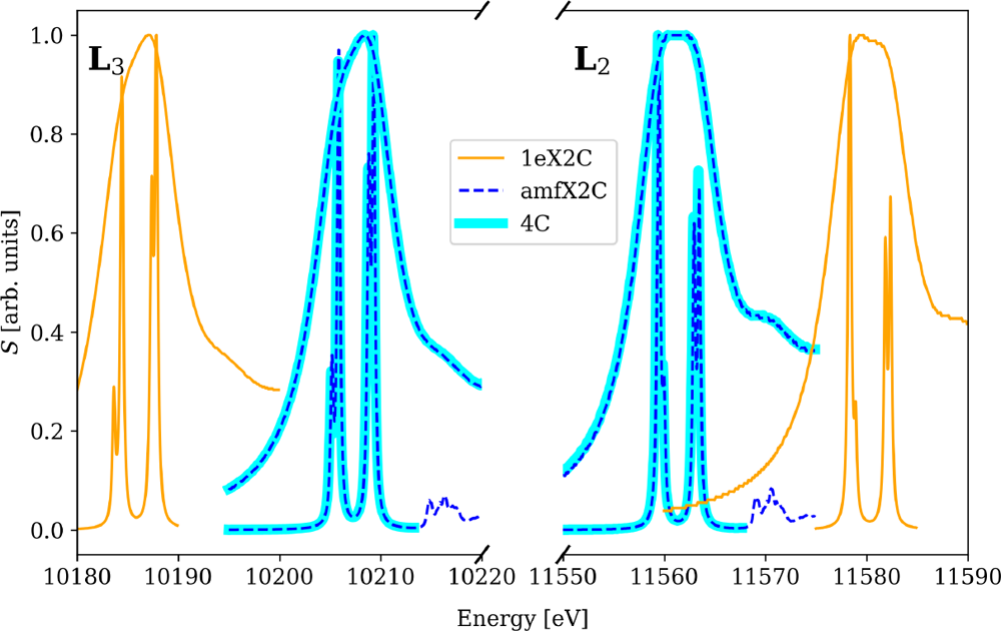
amfX2C Hamiltonian reproduces the 4c reference while 1eX2C Hamiltonian overestimates spin–orbit splitting leading to 20 eV shifts in XAS spectra of [WCl_4_(PMePh_2_)_2_] near W L_2,3_-edges calculated using PBE0-60HF functional and DZ/aDZ basis sets. Broad peaks were obtained with damping parameter γ = 3.0 eV, while narrow ones were obtained with γ = 0.15 eV.

**Figure 5:**
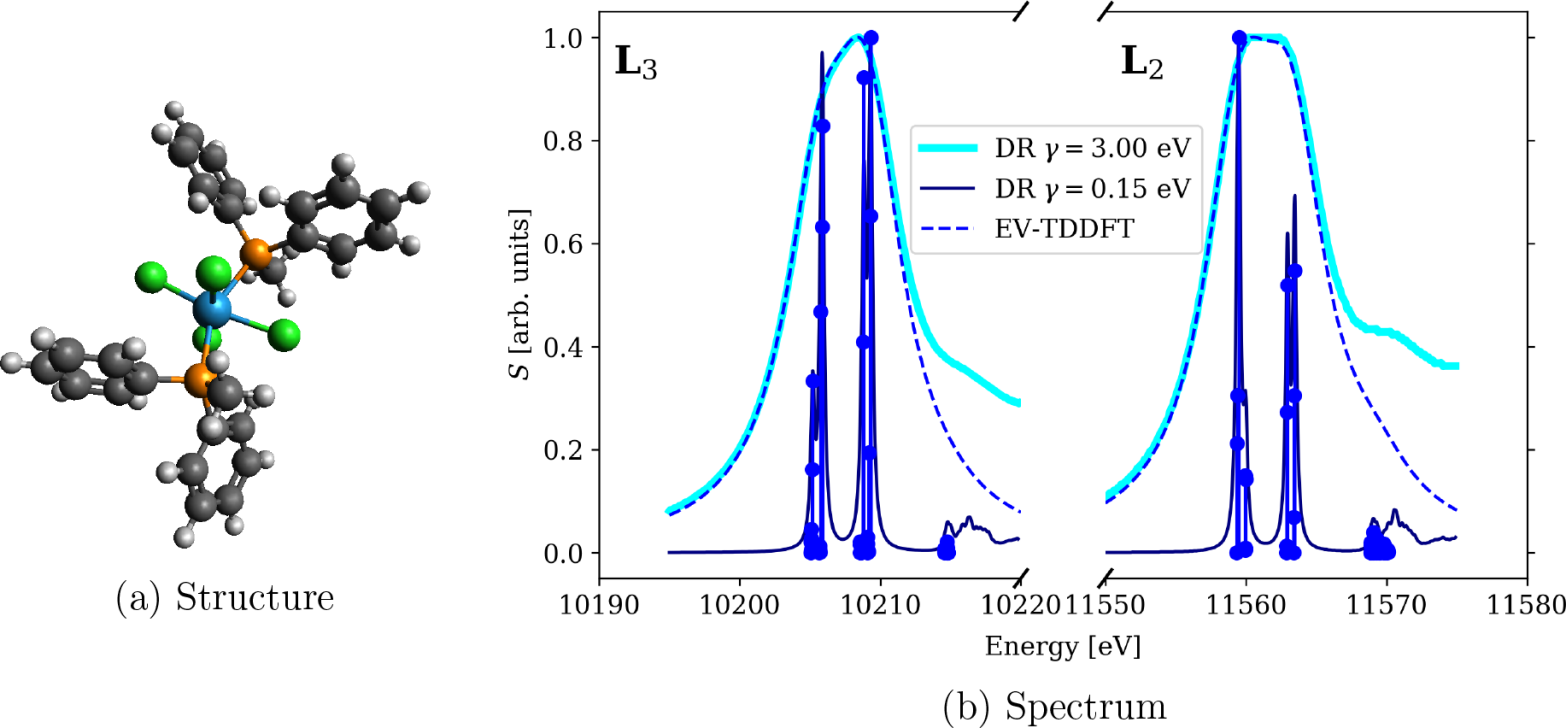
Comparison of damped response (DR) and eigenvalue (EV) TDDFT for XAS spectra of [WCl_4_(PMePh_2_)_2_] near W L_2,3_-edges calculated using PBE0-60HF functional and DZ/aDZ basis sets.

Second, we calculate XAS spectra near tungsten L_2,3_-edges of [WCl_4_(PMePh_2_)_2_] (Figure 5a) at the amfX2C relativistic level of theory. In addition to reproducing the 4c DR-TDDFT results, we also document the failure of 1eX2C in Figure 4 similarly as in Figure 2 for WCl_6_. The conclusion is the same: 1eX2C is unable to reproduce the reference 4c results due to its overestimation of spin–orbit splitting. Moreover, we include the calculation of excitation energies and transition moments using eigenvalue TDDFT. The final spectra are shown in Figure 5b, and the calculated eigenvalues and corresponding oscillator strengths are reported in Tables S2 and S3. The comparison of DR-TDDFT and EV-TDDFT showcases the pros and cons of these linear response TDDFT approaches. On the one hand, DR-TDDFT gives the full spectral function on the frequency interval of interest for a given damping parameter γ, which allows the experimental spectra to be reproduced. However, in order to resolve the broad peaks into individual transitions, one has to decrease the damping parameter. While this can yield fruitful results and interpretation of spectra (see our analysis of this system at the 4c level of theory in ref (85)), the EV-TDDFT accesses individual transitions directly, essentially in the limit γ → 0. On the other hand, in the iterative solution of EV-TDDFT, the user-defined number of eigenvalues is calculated from the lowest for the edge specified by the core–valence separation. This explains why the two methods initially lead to the same spectral function but start to depart for higher energies: more than the 50 transitions considered in EV-TDDFT would have been needed to match the spectra. The higher number of eigenvalues increases the computational cost of the method and puts strains on the stability of the iterative solver. The choice of the best-suited method thus depends on the chemical problem at hand, and this example showcases that the amfX2C-based implementation of both DR- and EV-TDDFT in the ReSpect program is up to the task of calculating XAS spectra of large complexes with heavy metal central atoms.

**Figure 6:**
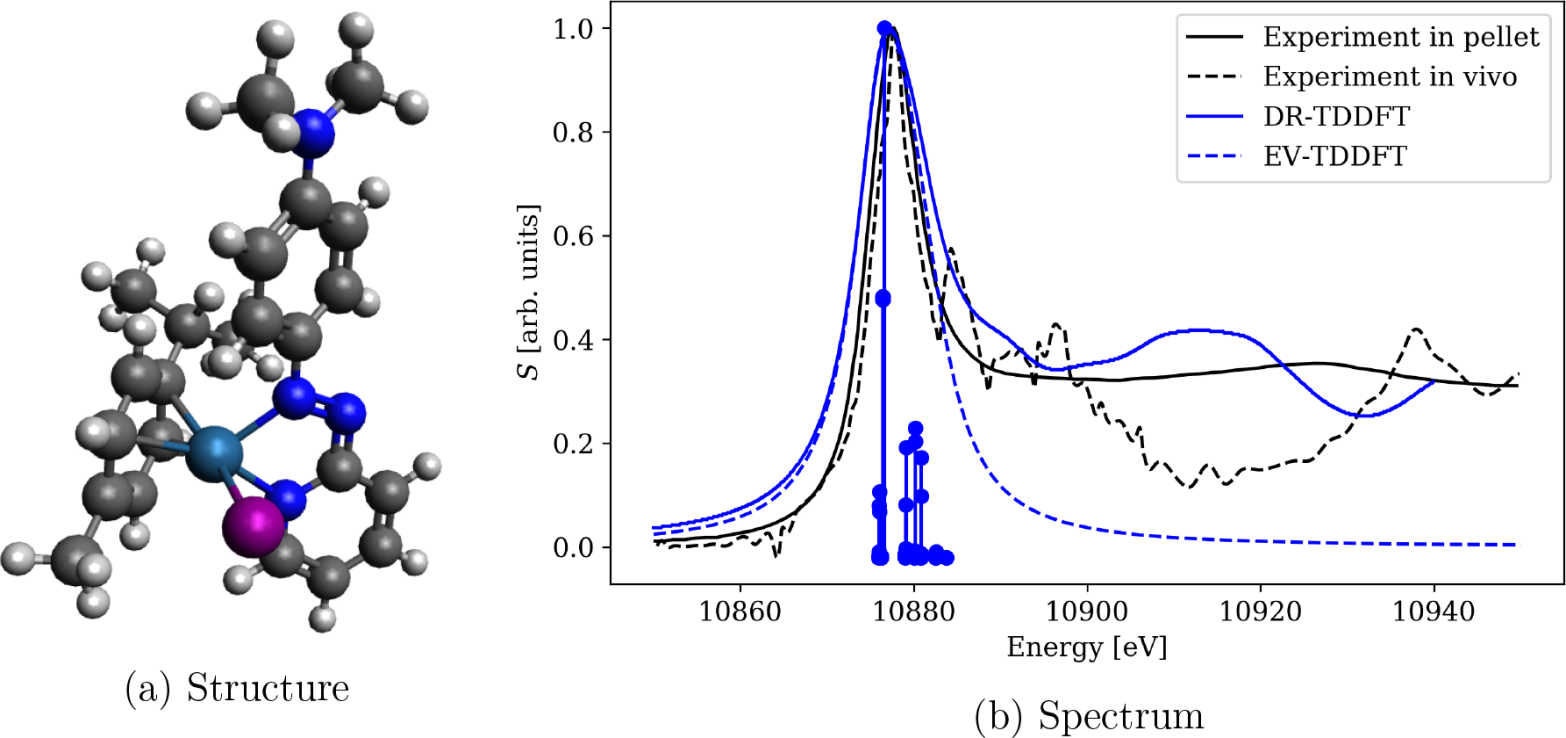
Comparison of experimental and theoretical damped response (DR) and eigenvalue (EV) TDDFT XAS spectra of [(η^6^-*p*-cym)Os(Azpy-NMe_2_)I]^+^ near Os L_3_-edge calculated using PBE0-60HF functional and DZ/aDZ basis sets. No shift was applied on the energy axis to manually align the spectra. Experimental spectra digitized from Ref. 97.

In the final application we focus on a system not considered in our previous work, [(η^6^-*p*-cym)Os(Azpy-NMe_2_)I]^+^, that also contains the heavy iodine atom as a ligand in addition to the large organic ligands; see Figure 6a. The final spectra are shown in Figure 6b, and the calculated eigenvalues and corresponding oscillator strengths are reported in Table S4. The system was investigated experimentally by Sanchez-Cano et al.^97^ as an anticancer drug, and its XAS spectra near the Os L_3_-edge were recorded both inside a cellulose pellet and in a cell culture. Both spectra are dominated by a major peak centered at 10 878 eV. The main features of the spectrum are reproduced in the amfX2C calculation for the molecule *in vacuo*. However, the reproduction of the satellite signals would require a detailed study of environmental effects which is beyond the scope of the present work. In this example, the combination of relativistic level of theory and the reparametrized PBE0-60HF xc potential achieved a staggering alignment of the spectra on the energy axis where *no additional shift was applied* to manually align the spectra.

## 5 Conclusions

We have presented a detailed theory derivation of X2C-based damped response time-dependent density functional theory (DR-TDDFT) and eigenvalue TDDFT (EV-TDDFT) starting from the four-component (4c) time-dependent Dirac–Kohn–Sham equations. We showed that X2C models known from time-independent calculations including 1eX2C as well as mmfX2C and the recently introduced (e)amfX2C can be extended to the time domain, and we derived the time-dependent Fock matrices that for (e)amfX2C include the important two-electron picture-change effects. We showed how initially time- and external-field-dependent X2C transformation matrices can be considered static for a weak field in the dipole approximation, which allowed us to formulate linear response TDDFT in the damped response and eigenvalue formalisms, where the final equations in molecular orbital basis have the same form and properties as the 4c equations. This in turn enabled a straightforward extension of the solvers previously developed at the 4c level of theory to these new two-component Hamiltonians.

We presented benchmark results for XAS spectra of transition metal and actinide compounds at the metal L- and M-edges, where spin–orbit (SO) splitting dominates the spectra. While the 1eX2C method overestimated the SO splitting, mmfX2C and (e)amfX2C shined. The latter X2C approaches fully reproduced the reference 4c results with a considerable saving of CPU time. This agreement allowed us to use the computational protocol for XAS calculations optimized at the 4c level of theory to be reused in X2C calculations. Since the assumption in 4c calculations was that relativistic effects were solved by such a high-level relativistic treatment, the same can be said about mmfX2C and (e)amfX2C calculations. This is contrasted with 1eX2C, where a new computational protocol relying on error cancellation would have to be developed.

As final highlights we presented calculated XAS spectra of large molecular systems involving about 50 atoms: [RuCl_2_(DMSO)_2_(Im)_2_] (Im = imidazole, DMSO = dimethyl sulfoxide), [WCl_4_(PMePh_2_)_2_] (Ph = phenyl), and [(η^6^-*p*-cym)Os(Azpy-NMe_2_)I]^+^ (*p*-cym = *p*-cymene, Azpy-NMe_2_ = 2-(*p*-([dimethylamino]phenylazo)pyridine)). We obtained an excellent agreement with experimental reference data in terms of both excitation energies and line shapes.

On the basis of the presented results, demonstrating both accuracy and computational efficiency, we envision the amfX2C and eamfX2C DR- and EV-TDDFT, where the full calculations including the initial SCF are performed in a two-component regime, to become a new paradigm for relativistic calculations of XAS spectra of large molecules containing heavy elements.

## Appendix A: Limiting Behavior of the X2C Unitary Transformation

In this section we prove the formulas for estimation of the time and field derivatives of the X2C unitary transformation when the system is in the presence of an external oscillating field of frequency ω and amplitude . The estimation expressions can be written as

54

55

where we used a notation that distinguishes between field-dependent, *x*^(*n*^(*t*), and field-independent, *x*_*u*_^(*n*)^(*t*), variables as defined in the following perturbational expression:

56

57

In the subsequent discussion it becomes advantageous to expand the X2C unitary transformation^22^

58

in its Taylor series around **R** = 0

59

where the brackets ∥·∥ represent some suitable matrix norm, e.g., Frobenius norm. Here and in the following we employ a simplified notation for the coupling matrix, . From eq 59 it is clear that, to estimate the linear response of the matrix , it is sufficient to analyze the behavior of the coupling matrix **R**. To obtain the limiting behavior of **R**, one may explore its dependence on time, field, and speed of light *c*.

In the next paragraph, we analyze the limiting behavior of **R** with respect to *c*. For that purpose, we may utilize the definition of the X2C unitary transformation presented in eq 12, where the off-diagonal—LS and SL—blocks of the transformed matrix are set to zero. However, a more transparent alternative is to use the expression for determining the coupling matrix within the one-step X2C method; see eq 23 in ref (20) or eq 8 in ref (22).

60

with **C**_+_ being the 4c positive-energy MO coefficients. If one uses orthonormal basis, then **C**_+_^L^ and **C**_+_^S^ are of order *c*^0^ and *c*^–1^, respectively. If we assume that the left-hand side of eq 60 has a convergent Laurent series with *c* as the variable, then the condition  is satisfied for each order of this series separately. By analyzing these conditions order by order, it follows that the first nonzero term in the expansion of the coupling matrix is of order *c*^–1^ and therefore its limiting behavior can be written as

61

To further improve the estimation of the coupling matrix, one can explore its time and field dependence. If the molecular system is subject to the time-dependent external electric field, **E**(*t*), the time-dependent Kohn–Sham equation can be viewed as a representation of a continuous time-invariant system that assigns the output signal—induced electric dipole moment, X2C unitary transformation, and other time-dependent quantities—to the input signal, **E**(*t*). By choosing the harmonic time dependence of the electric field, , the coupling matrix **R**, its time derivative, and its linear response, can be expressed using the Volterra series as follows:

62

63

64

Combining eqs 61–63, one can write the limiting behavior of the coupling matrix as

65

66

Finally, to prove eqs 54 and 55, one simply uses the linear part of the expansion in eq 59 and the estimations for the coupling matrix **R** in eqs 65 and 66. *Q.E.D.*

## Appendix B: Contribution of the Negative-Energy States to Two-Component EOM

The transformation of the four-component EOM, eq 1, by the X2C unitary matrix **U** results in two separate two-component equations [see eq 15]. However, these equations are still coupled, because, for example, the LL equation whose solutions are  depends on the solutions of the SS equation, , through the density matrix . In addition, the complex linear electric dipole polarizability tensor **α**(ω), eq 51, whose imaginary part determines the electronic absorption spectrum [eq 49], depends on both solutions  and . Therefore, in contrast to the static case, in the time domain it does not suffice to fully transform 4c EOM to one 2c EOM to find the X2C unitary transformation; one rather needs to solve two 2c EOMs. However, thanks to the X2C unitary transformation, the large component (small component) of the time- and field-dependent occupied MO coefficients, , depend only on the positive-energy (negative-energy) reference MOs, ***C̃***_+_ (***C̃***_–_) [see eq 16]

67

The contribution of the negative-energy states to the complex polarizability tensor **α** is only of order *c*^–4^ and can thus safely be neglected. Equation 15 is then fully decoupled, and it becomes sufficient to solve only one 2c EOM, eq 17, to obtain the complex polarizability tensor and resulting electronic absorption spectrum.

To prove that the effect of the negative-energy states in the complex polarizability tensor is of order *c*^–4^, one can simply examine the expressions for the tensor **α**, eq 51, and DR-TDDFT equations, eq 47. Note that we get the same result regardless of whether we use the two- or four-component picture for the proof. One can then employ a bit simpler four-component equation, where in the orthonormal basis it holds that

68

69

as *c* → ∞. Based on the DR-TDDFT equation, eq 47, one can then estimate the response coefficients for the negative-energy states as *X*_– +_ = *Y*_– +_ = *O*(*c*^–3^) and their contribution to the complex polarizability tensor, eq 51, as *O*(*c*^–4^). *Q.E.D.*

## Supporting Information Available

Molecular geometries, EV-TDDFT, and eamfX2C DR-TDDFT results (PDF)
