# Supplementary material for: Exact Two-Component TDDFT with Simple Two-Electron Picture-Change Corrections: X-ray Absorption Spectra Near L- and M-Edges of Four-Component Quality at Two-Component Cost
Source: J Phys Chem A. 2023 Feb 1;127(5):1360–76. doi: 10.1021/acs.jpca.2c08307 (PMC9923756; doi:10.1021/acs.jpca.2c08307)
Supplement: Supplementary file 1 — jp2c08307_si_001.pdf [file jp2c08307_si_001.pdf]

Exact Two-Component TDDFT with Simple Two-Electron Picture-Change Corrections: X-ray Absorption Spectra near L- and M-Edges of Four-Component Quality at Two-Component Cost

Supporting information

Lukas Konecny,[*,†,‡] Stanislav Komorovsky,[¶] Jan Vicha,[§] Kenneth Ruud,[†,‖] and Michal Repisky[*,†,⊥]

†*Hylleraas Centre for Quantum Molecular Sciences, Department of Chemistry, UiT The Arctic University of Norway, N-9037 Tromsø, Norway*

‡*Max Planck Institute for the Structure and Dynamics of Matter, Center for Free Electron Laser Science, Luruper Chaussee 149, 22761 Hamburg, Germany*

¶*Institute of Inorganic Chemistry, Slovak Academy of Sciences, Dúbravská cesta 9, SK-84536 Bratislava, Slovakia*

§*Centre of Polymer Systems, University Institute, Tomas Bata University in Zlín, CZ-76001 Zlín, Czech Republic*

‖*Norwegian Defence Research Establishment, P.O. Box 25, 2027 Kjeller, Norway*

⊥*Department of Physical and Theoretical Chemistry, Faculty of Natural Sciences, Comenius University, Ilkovicova 6, SK-84215 Bratislava, Slovakia*

E-mail: lukas.konecny@uit.no; michal.repisky@uit.no

S1 Extended atomic mean-field X2C

Table S1: Main line positions and spin–orbit splittings in XAS spectra calculated using DR-TDDFT with PBE0 and PBE0-xHF functionals in Dyall-DZ/aDZ basis sets and amfX2C and eamfX2C Hamiltonians compared with experimental data.

		Exp.[a]	PBE0-25HF		PBE0-xHF		
			amfX2C	eamfX2C	xHF	amfX2C	eamfX2C
$VOCl_3$	L_3	516.9	507.5	507.5	50	515.2	515.2
	L_2	523.8	514.2	514.2	50	522.0	522.0
	ΔSO	6.9	6.7	6.7		6.8	6.8
CrO_2Cl_2	L_3	579.9	571.4	571.4	50	580.0	580.0
	L_2	588.5	579.5	579.5	50	588.2	588.2
	ΔSO	8.6	8.1	8.1		8.2	8.2
${MoS_4}^{2-}$	L_3	2521.7	2489.3	2489.3	60	2523.1	2523.1
	L_2	2626.0	2595.9	2595.9	60	2627.6	2627.6
	ΔSO	104.3	106.6	106.6		104.5	104.5
	M_5	228.7	225.4	225.4	40	228.9	228.9
	M_4	231.7	228.7	228.7	40	232.2	232.2
	ΔSO	3.0	3.3	3.3		3.3	3.3
${PdCl_6}^{2-}$	L_3	3177.8	3138.2	3138.2	60	3173.4	3173.4
	L_2	3334.7	3297.4	3297.4	60	3332.9	3332.9
	ΔSO	156.9	159.2	159.2		159.5	159.5
WCl_6	L_3	10212.2	10139.9	10139.9	60	10207.3	10207.3
	L_2	11547.0	11492.7	11492.7	60	11561.6	11561.6
	ΔSO	1334.8	1352.8	1352.8		1354.3	1354.3
${ReO_4}^{-}$	L_3	10542.0	10471.7	10471.7	60	10541.0	10541.0
	L_2	—	11911.9	11911.9	60	11982.1	11982.1
	ΔSO	—	1440.2	1440.2		1441.1	1441.1
$UO_2(NO_3)_2$	M_5	—	3515.3	3515.3	60	3549.9	3549.9
	M_4	3727.0	3693.2	3693.2	60	3728.0	3728.0
	ΔSO	—	177.9	177.9		178.1	178.1

S2 $[RuCl_2(DMSO)_2(Im)_2]$ spectrum with applied shifts

Figure S1: XAS spectrum of $[RuCl_2(DMSO)_2(Im)_2]$ near overlapping Cl K-edge and Ru L_3-edge calculated using PBE0-60HF functional and DZ/aDZ basis sets. Both absorption edges are reproduced sufficiently well when different inverse lifetimes (γ) of core-excited states of different atoms are accounted for. Edge-specific energy shifts ($\Delta\omega$) were applied to align the calculated spectra with the experiment to ease line shape comparison.

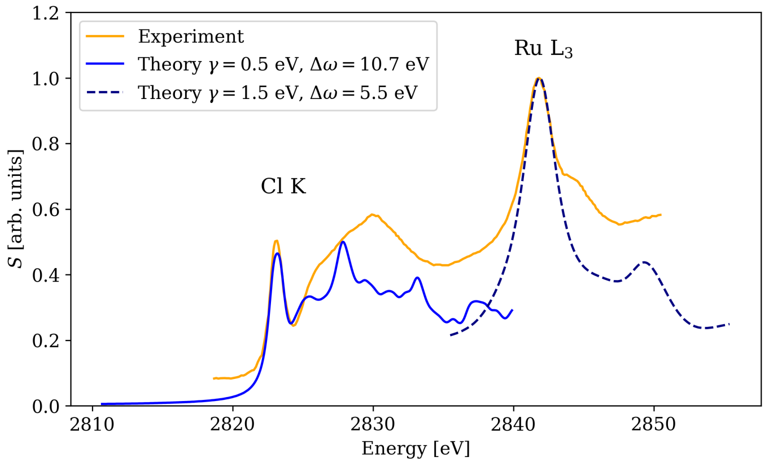

S3 EV-TDDFT results

Table S2: $[WCl_4(PMePh_2)_2]$, W L_3-edge, excitation energies ($\hbar\omega_N$) and oscillator strengths (f_N [dimensionless]) calculated with PBE0-60HF functional in Dyall-DZ/aDZ basis sets and amfX2C EV-TDDFT.

N	$\hbar\omega_N$ [eV]	$\hbar\omega_N$ [au]	f_N	N	$\hbar\omega_N$ [eV]	$\hbar\omega_N$ [au]	f_N
1	10205.0372	375.02821793	0.0000000802	26	10209.0802	375.17679454	0.0000153073
2	10205.0612	375.02909802	0.0000185476	27	10209.0897	375.17714389	0.0000267772
3	10205.0739	375.02956589	0.0000394456	28	10209.1481	375.17928882	0.0000016705
4	10205.0877	375.03007152	0.0000039289	29	10209.1494	375.17933606	0.0000013196
5	10205.0898	375.03014869	0.0000234391	30	10209.2014	375.18124923	0.0001702869
6	10205.1242	375.03141254	0.0000022708	31	10209.3018	375.18493799	0.0005752963
7	10205.1672	375.03299427	0.0001423355	32	10209.3419	375.18641202	0.0008803732
8	10205.1915	375.03388654	0.0002930653	33	10214.5554	375.37800492	0.0000001418
9	10205.6280	375.04992703	0.0000000400	34	10214.5557	375.37801570	0.0000015334
10	10205.6538	375.05087719	0.0000075170	35	10214.5579	375.37809636	0.0000035827
11	10205.6681	375.05139972	0.0000064811	36	10214.5587	375.37812330	0.0000063935
12	10205.6948	375.05238368	0.0000116472	37	10214.5916	375.37933565	0.0000011032
13	10205.7037	375.05271123	0.0000008806	38	10214.5922	375.37935507	0.0000003479
14	10205.7383	375.05398073	0.0004109804	39	10214.5930	375.37938502	0.0000049488
15	10205.8558	375.05829920	0.0005560893	40	10214.5982	375.37957714	0.0000063643
16	10205.9067	375.06016939	0.0007288985	41	10214.7282	375.38435340	0.0000002075
17	10208.5546	375.15747978	0.0000001677	42	10214.7283	375.38435711	0.0000002480
18	10208.5621	375.15775551	0.0000132690	43	10214.7335	375.38455033	0.0000183634
19	10208.5732	375.15816081	0.0000184993	44	10214.7341	375.38457202	0.0000073203
20	10208.6230	375.15999371	0.0000000917	45	10214.7664	375.38575953	0.0000001696
21	10208.6243	375.16003940	0.0000024153	46	10214.7684	375.38583317	0.0000075231
22	10208.6560	375.16120358	0.0000140245	47	10214.7695	375.38587360	0.0000025197
23	10208.7737	375.16552929	0.0003601721	48	10214.7726	375.38598657	0.0000019063
24	10208.8324	375.16768758	0.0008113329	49	10214.7923	375.38670897	0.0000002744
25	10209.0728	375.17652029	0.0000000048	50	10214.7923	375.38671024	0.0000003063

Table S3: $[WCl_4(PMePh_2)_2]$, W L_2-edge, excitation energies ($\hbar\omega_N$) and oscillator strengths (f_N [dimensionless]) calculated with PBE0-60HF functional in Dyall-DZ/aDZ basis sets and amfX2C EV-TDDFT.

N	$\hbar\omega_N$ [eV]	$\hbar\omega_N$ [au]	f_N	N	$\hbar\omega_N$ [eV]	$\hbar\omega_N$ [au]	f_N
1	11559.3432	424.79804531	0.0000000385	26	11569.1008	425.15663070	0.0000094144
2	11559.3739	424.79917343	0.0001141672	27	11569.1022	425.15668222	0.0000007180
3	11559.3973	424.80003554	0.0001639714	28	11569.1026	425.15669855	0.0000005860
4	11559.5075	424.80408466	0.0005377165	29	11569.2942	425.16373704	0.0000000601
5	11559.9485	424.82029098	0.0000027035	30	11569.2948	425.16376102	0.0000004731
6	11559.9697	424.82107118	0.0000047387	31	11569.2976	425.16386261	0.0000139448
7	11559.9757	424.82128880	0.0000804506	32	11569.2999	425.16394874	0.0000094957
8	11559.9819	424.82151668	0.0000767305	33	11569.4925	425.17102594	0.0000001145
9	11562.8686	424.92760252	0.0000000245	34	11569.4932	425.17105310	0.0000001776
10	11562.8864	424.92825456	0.0000069729	35	11569.4947	425.17110504	0.0000032347
11	11562.9336	424.92999171	0.0001466388	36	11569.4951	425.17112216	0.0000064364
12	11562.9681	424.93125790	0.0002790122	37	11569.7891	425.18192624	0.0000001030
13	11563.3875	424.94667157	0.0000000256	38	11569.7904	425.18197194	0.0000004880
14	11563.4124	424.94758661	0.0000368830	39	11569.7910	425.18199670	0.0000091427
15	11563.4491	424.94893410	0.0001637669	40	11569.7953	425.18215519	0.0000020344
16	11563.4861	424.95029494	0.0002941724	41	11569.9156	425.18657368	0.0000000347
17	11568.8609	425.14781608	0.0000001657	42	11569.9167	425.18661407	0.0000010242
18	11568.8613	425.14783159	0.0000000228	43	11569.9170	425.18662550	0.0000045266
19	11568.8639	425.14792362	0.0000073038	44	11569.9184	425.18667829	0.0000006685
20	11568.8691	425.14811787	0.0000103740	45	11570.0416	425.19120482	0.0000000629
21	11569.0343	425.15418796	0.0000000232	46	11570.0417	425.19121021	0.0000003073
22	11569.0380	425.15432486	0.0000209533	47	11570.0421	425.19122466	0.0000009947
23	11569.0388	425.15435234	0.0000007989	48	11570.0424	425.19123529	0.0000034394
24	11569.0393	425.15436989	0.0000005691	49	11570.1195	425.19406667	0.0000003977
25	11569.0975	425.15650949	0.0000000616	50	11570.1218	425.19415411	0.0000009060

Table S4: $[(\eta^6\text{−p−cym})\text{Os}(\text{Azpy−NMe}_2)\text{I}]^+$, Os L_3-edge, excitation energies ($\hbar\omega_N$) and oscillator strengths (f_N [dimensionless]) calculated with PBE0-60HF functional in Dyall-DZ/aDZ basis sets and amfX2C EV-TDDFT.

N	$\hbar\omega_N$ [eV]	$\hbar\omega_N$ [au]	f_N	N	$\hbar\omega_N$ [eV]	$\hbar\omega_N$ [au]	f_N
1	10875.9058	399.68218363	0.0000000492	26	10880.0508	399.83451133	0.0000003496
2	10875.9195	399.68268889	0.0000019078	27	10880.0799	399.83558005	0.0000167895
3	10875.9363	399.68330450	0.0000032528	28	10880.0831	399.83569542	0.0000137384
4	10875.9683	399.68447914	0.0000135473	29	10880.0952	399.83614008	0.0000004081
5	10875.9852	399.68510287	0.0000061438	30	10880.1107	399.83671179	0.0000016162
6	10876.0164	399.68624876	0.0001226762	31	10880.1730	399.83900159	0.0002761370
7	10876.0442	399.68727093	0.0001088804	32	10880.1831	399.83937011	0.0003079550
8	10876.0647	399.68802486	0.0001568152	33	10880.8021	399.86211830	0.0000000122
9	10876.2172	399.69362745	0.0000001761	34	10880.8023	399.86212727	0.0000004302
10	10876.2273	399.69399685	0.0000033202	35	10880.8160	399.86263161	0.0000094434
11	10876.2366	399.69433889	0.0000001852	36	10880.8261	399.86300148	0.0000085505
12	10876.2390	399.69442918	0.0000036621	37	10880.8344	399.86330507	0.0000000205
13	10876.2521	399.69490943	0.0000001779	38	10880.8465	399.86375154	0.0000075638
14	10876.3982	399.70028069	0.0006136716	39	10880.8761	399.86483826	0.0001465249
15	10876.4338	399.70158657	0.0006215861	40	10880.8883	399.86528732	0.0002383096
16	10876.5993	399.70766861	0.0012608493	41	10882.5427	399.92608714	0.0000000009
17	10879.0144	399.79642284	0.0000000471	42	10882.5428	399.92608770	0.0000000629
18	10879.0176	399.79654203	0.0000047814	43	10882.5434	399.92611195	0.0000002862
19	10879.0206	399.79665063	0.0000099618	44	10882.5465	399.92622374	0.0000098197
20	10879.0428	399.79746819	0.0000004920	45	10882.5562	399.92658077	0.0000000022
21	10879.0434	399.79748900	0.0000000410	46	10882.5565	399.92659080	0.0000000574
22	10879.0595	399.79807990	0.0000219663	47	10882.5577	399.92663531	0.0000060947
23	10879.0901	399.79920618	0.0001265247	48	10882.5618	399.92678708	0.0000133444
24	10879.1107	399.79996226	0.0002626778	49	10883.7326	399.96981288	0.0000000383
25	10880.0508	399.83450818	0.0000000750	50	10883.7326	399.96981395	0.0000000605

S4 Geometries

All molecular geometries are given in Ångström.

Table S5: Molecular geometry of $VOCl_3$.

Atom	x	y	z
V	2.2290187	-1.1797130	-0.0908750
O	2.4041704	-1.0461917	-1.6126195
Cl	0.2242083	-0.6442191	0.3979482
Cl	2.6220625	-3.2042145	0.4493642
Cl	3.6134815	0.1359875	0.8561821

Table S6: Molecular geometry of CrO_2Cl_2.

Atom	x	y	z
Cr	0.88078158	-0.13245116	-0.02959505
Cl	-1.22227445	-0.14808946	-0.05647850
Cl	1.61111615	0.85367740	1.67858777
O	1.39666198	-1.58039095	-0.03621237
O	1.39682473	0.58566316	-1.28685686

Table S7: Molecular geometry of $MoS_4{}^{2-}$.

Atom	x	y	z
Mo	2.4310175	1.7387470	7.0093109
S	2.7198499	1.7387524	4.8370074
S	3.3598198	-0.0499583	7.8694376
S	0.2866474	1.7387525	7.4614107
S	3.3598163	3.5274565	7.8694344

Table S8: Molecular geometry of WCl_6.

Atom	x	y	z
W	3.5315869	2.0389600	5.7670454
Cl	5.1495989	2.9730442	4.4458810
Cl	3.5315673	0.1706769	4.4458067
Cl	3.5315673	3.9072434	7.0882847
Cl	5.1495990	1.1048756	7.0882105
Cl	1.9135701	1.1048759	7.0882103
Cl	1.9135703	2.9730440	4.4458812

Table S9: Molecular geometry of $PdCl_6{}^{2-}$.

Atom	x	y	z
Pd	3.5315810	2.0389600	5.7670462
Cl	5.1848568	2.9932265	4.4169306
Cl	3.5315777	0.1301751	4.4167597
Cl	3.5315774	3.9477441	7.1173315
Cl	5.1848559	1.0846937	7.1171605
Cl	1.8783060	1.0846939	7.1171607
Cl	1.8783051	2.9932267	4.4169307

Table S10: Molecular geometry of $ReO_4{}^{-}$.

Atom	x	y	z
Re	1.4934685	-0.5338437	0.8411442
O	1.6119799	0.5314972	2.1938310
O	0.2220604	-0.0041968	-0.1988355
O	2.9853466	-0.5297356	-0.0263973
O	1.1555746	-2.1330311	1.3952877

Table S11: Molecular geometry of $UO_2(NO_3)_2$.

Atom	x	y	z
U	3.4762959	0.5300554	-0.5098080
N	2.9333958	0.4442629	2.2755600
N	4.0151241	0.6301142	-3.2951080
O	5.1825442	0.4323044	-0.1801379
O	1.7699108	0.6261756	-0.8395763
O	3.1200485	1.5344677	1.6308641
O	3.9361036	1.6737949	-2.5577927
O	2.7066333	0.4085578	3.4373390
O	3.0150766	-0.6036928	1.5447681
O	3.8324350	-0.4638204	-2.6558319
O	4.2358920	0.6727204	-4.4578064

Table S12: Molecular geometry of $[RuCl_2(DMSO)_2(Im)_2]$.

Atom	x	y	z
Ru	1.0823808	1.3041079	0.1880267
Cl	0.7734561	3.6018235	0.8740608
Cl	1.1450420	2.0186901	-2.1182100
C	-1.6567654	0.0998657	-1.4047446
S	-1.1656750	1.0593649	0.0196562
C	-1.9340021	2.5986308	-0.4414959
O	-1.9101692	0.5196407	1.1700987
H	-1.3252009	-0.9200840	-1.2193946
H	-1.1795748	0.5246730	-2.2873559
H	-2.7434568	0.1438438	-1.4709946
H	-1.4323182	2.9790320	-1.3304312
H	-1.7821491	3.2881488	0.3841397
H	-2.9904420	2.3920068	-0.6109234
C	3.0308443	-1.4108433	0.2858381
S	1.4733775	-0.8016002	-0.3646613
C	1.8242665	-1.0940299	-2.0948804
O	0.4878784	-1.8368448	0.0261280
H	2.9634280	-1.3531521	1.3707434
H	3.8599065	-0.8055382	-0.0738677
H	3.1273334	-2.4504412	-0.0258069
H	2.6482235	-0.4590574	-2.4128611
H	0.9382740	-0.8169022	-2.6591589
H	2.0454381	-2.1555655	-2.2038337
N	4.8832852	3.0975981	0.0694091
C	5.1759831	2.3110140	1.1518847
C	4.0826615	1.5223009	1.3294467
N	3.1451298	1.8185636	0.3724159
C	3.6539808	2.7806910	-0.3664411
H	6.1061345	2.3803382	1.6874496
H	3.9070607	0.7843888	2.0917226
H	3.1420966	3.2473721	-1.1920172
H	5.4663956	3.8150351	-0.3215818
N	0.4578474	-0.1818650	4.0845028
C	1.0817823	0.9868169	4.4389115
C	1.4131809	1.5947223	3.2697006
N	1.0010208	0.8071012	2.2216234
C	0.4209525	-0.2533658	2.7452230
H	1.2189723	1.2831078	5.4635236
H	1.8673423	2.5536744	3.0935703
H	-0.0380690	-1.0482396	2.1807274
H	0.0506571	-0.8546136	4.7078172

Table S13: Molecular geometry of $[WCl_4(PMePh_2)_2]$.

Atom	*x*	*y*	*z*	Atom	*x*	*y*	*z*
W	2.1396762	4.2841474	6.1647819	C	2.9271611	-0.9579594	5.5654926
Cl	1.7432161	3.7830972	8.4996792	C	3.8414400	1.1251025	4.7999230
Cl	2.4984758	4.6486696	3.8172660	C	3.3650318	-0.1444191	4.5295200
Cl	0.5911128	2.7270541	5.6001838	H	0.9682672	9.5992083	10.8052290
Cl	3.4956600	6.1846445	6.7347833	H	1.5878126	8.9167095	6.6373039
P	0.5458535	6.1954535	6.2737679	H	1.7666026	10.3765651	8.5953638
P	4.3461574	3.3042894	6.4232453	H	-0.0087443	7.3358963	11.0289461
C	0.6646963	7.2969609	7.7117245	H	-0.1903295	5.8707784	9.0729657
C	1.2228012	8.5665383	7.5930552	H	-1.8661987	7.0788948	4.8053713
C	1.3295597	9.3913517	8.7014390	H	-4.2137335	6.3912794	4.9336621
C	0.8843765	8.9547733	9.9387678	H	-4.9050855	4.6042765	6.4981448
C	0.3345871	7.6874687	10.0640832	H	-3.2096541	3.4899772	7.9164816
C	0.2256896	6.8613490	8.9592635	H	-0.8446056	4.1442594	7.7612522
C	-1.1952714	5.6734336	6.2921825	H	0.4551793	6.7211631	3.9518795
C	-2.1504634	6.2931429	5.4922833	H	1.7582850	7.5755335	4.7607722
C	-3.4798428	5.9062597	5.5652632	H	0.0858605	8.1773708	4.9353337
C	-3.8661818	4.9047461	6.4413484	H	6.7250372	1.7951529	5.8465279
C	-2.9167602	4.2816982	7.2383004	H	8.6741577	2.1986178	4.4002772
C	-1.5870293	4.6561451	7.1604488	H	8.7870891	4.2501892	3.0201927
C	0.7108913	7.2899733	4.8441461	H	6.9427447	5.8934081	3.1030360
C	5.7370639	3.6286595	5.3056270	H	4.3480672	3.1402579	8.8189435
C	6.7786429	2.7025010	5.2557681	H	5.4105744	4.4185146	8.2223403
C	7.8718475	2.9250331	4.4375089	H	5.9572413	2.7238084	8.1293803
C	7.9333789	4.0761702	3.6637900	H	3.4332517	1.1369858	8.1708822
C	6.9003466	4.9981780	3.7107671	H	2.6159478	-1.1321816	7.6821575
C	5.8013669	4.7786234	4.5281046	H	2.5472227	-1.9491450	5.3504252
C	5.0951779	3.3869051	8.0671062	H	3.3251641	-0.4965786	3.5064514
C	2.9667588	-0.5012079	6.8751226	H	4.1576888	1.7740156	3.9923629
C	3.8904257	1.5838555	6.1162153	H	4.9887507	5.4898614	4.5555525
C	3.4411405	0.7688828	7.1534064				

Table S14: **Molecular geometry of $[(\eta^6-p-cym)Os(Azpy-NMe_2)I]^+$.**

Atom	x	y	z	Atom	x	y	z
Os	1.97615074	8.18879987	3.19402535	C	1.01983320	9.74446192	4.56401148
I	0.33822012	8.91089372	1.16349441	C	0.34332604	8.48243303	4.65965422
N	3.48871236	8.64837074	1.90523361	H	-0.71437823	8.43589197	4.43636549
C	4.01567771	9.85854202	1.67939752	C	1.03336527	7.31535284	5.00332992
H	3.57716926	10.67971041	2.22781748	H	0.50286840	6.37379818	5.05028102
C	5.04671833	10.05115074	0.78953629	C	2.44953886	7.31477736	5.17270383
H	5.44394161	11.04711598	0.64745590	C	3.15070677	6.02504758	5.48337217
C	5.54858106	8.96337982	0.08130656	H	2.58137617	5.23588043	4.98277711
H	6.35601365	9.09493717	-0.62788227	C	3.08229179	5.78330667	6.98854183
C	4.99145764	7.72306237	0.28046240	H	2.05277292	5.78523995	7.35042828
H	5.31341023	6.84362562	-0.26009289	H	3.63312280	6.55659838	7.52846722
C	3.95107914	7.59451574	1.19677342	H	3.52676061	4.81797830	7.23400121
N	3.33425418	6.39780378	1.37411031	C	4.57980489	5.97351732	4.97639943
N	2.34848118	6.47105822	2.19086681	H	4.63812359	6.20461417	3.91140725
C	1.66141885	5.27520789	2.38130075	H	4.98762383	4.97394480	5.12857936
C	0.30319875	5.29053198	2.70588018	H	5.22520755	6.66724068	5.51958076
H	-0.21079489	6.24090739	2.76037782	C	0.26408533	10.98413333	4.24229440
C	-0.39435229	4.12086057	2.85332039	H	-0.47391729	10.79583605	3.46241760
H	-1.45446596	4.16844007	3.05479482	H	0.92805767	11.77853992	3.90445146
C	0.24560866	2.87034541	2.70191487	H	-0.25890288	11.32965197	5.13789916
N	-0.42960396	1.71542339	2.85684651	C	0.23225290	0.44833157	2.64052931
C	1.62236511	2.87255903	2.37173039	C	-1.83543906	1.73200360	3.19121256
H	2.15244843	1.93874895	2.25440614	H	-2.01239072	2.25973037	4.13169532
C	2.30226431	4.04483037	2.19439823	H	-2.18475660	0.70992652	3.30556820
H	3.35164560	4.03415166	1.93349393	H	-2.42773379	2.20923070	2.40492043
C	3.12588184	8.54446544	5.01320855	H	0.63045923	0.37615173	1.62478879
H	4.20537736	8.57680347	5.06203778	H	-0.48523979	-0.35476107	2.78157806
C	2.40559848	9.74646236	4.74023105	H	1.05147937	0.30111578	3.34966942
H	2.95283366	10.67041170	4.60177837				
